# Keeping the home fires burning^†^: AMP-activated protein kinase

**DOI:** 10.1098/rsif.2017.0774

**Published:** 2018-01-17

**Authors:** D. Grahame Hardie

**Affiliations:** Division of Cell Signalling and Immunology, School of Life Sciences, University of Dundee, Dow Street, Dundee, DD1 5EH, UK

**Keywords:** AMP-activated protein kinase, AMP-activated protein kinase, mitochondria, energy homeostasis, cell signalling, adenosine triphosphate

## Abstract

Living cells obtain energy either by oxidizing reduced compounds of organic or mineral origin or by absorbing light. Whichever energy source is used, some of the energy released is conserved by converting adenosine diphosphate (ADP) to adenosine triphosphate (ATP), which are analogous to the chemicals in a rechargeable battery. The energy released by the conversion of ATP back to ADP is used to drive most energy-requiring processes, including cell growth, cell division, communication and movement. It is clearly essential to life that the production and consumption of ATP are always maintained in balance, and the AMP-activated protein kinase (AMPK) is one of the key cellular regulatory systems that ensures this. In eukaryotic cells (cells with nuclei and other internal membrane-bound structures, including human cells), most ATP is produced in mitochondria, which are thought to have been derived by the engulfment of oxidative bacteria by a host cell not previously able to use molecular oxygen. AMPK is activated by increasing AMP or ADP (AMP being generated from ADP whenever ADP rises) coupled with falling ATP. Relatives of AMPK are found in essentially all eukaryotes, and it may have evolved to allow the host cell to monitor the output of the newly acquired mitochondria and step their ATP production up or down according to the demand. Structural studies have illuminated how AMPK achieves the task of detecting small changes in AMP and ADP, despite the presence of much higher concentrations of ATP. Recently, it has been shown that AMPK can also sense the availability of glucose, the primary carbon source for most eukaryotic cells, via a mechanism independent of changes in AMP or ADP. Once activated by energy imbalance or glucose lack, AMPK modifies many target proteins by transferring phosphate groups to them from ATP. By this means, numerous ATP-producing processes are switched on (including the production of new mitochondria) and ATP-consuming processes are switched off, thus restoring energy homeostasis. Drugs that modulate AMPK have great potential in the treatment of metabolic disorders such as obesity and Type 2 diabetes, and even cancer. Indeed, some existing drugs such as metformin and aspirin, which were derived from traditional herbal remedies, appear to work, in part, by activating AMPK.

## 1. Being alive requires a constant and rechargeable source of energy

The cell (a term coined by one of the founder members of the Royal Society, Robert Hooke, while making microscopic observations of plant material, which he thought looked ‘much like a honeycomb' [1]) is the fundamental unit of all living organisms. There are now believed to be three major domains of life: (i) the *archaea*,^1^ which inhabit extreme environments like hot springs and deep ocean vents and (as their name suggests) may be the most ancient life-form; (ii) the *bacteria*, and (iii) the *eukarya* or *eukaryotes*. Archaeal and bacterial cells range in diameter from 1 to 5 µm, and have few internal membranes, while eukaryotic cells are larger (typically 10–100 µm), envelop their DNA in a membrane-bound nucleus and contain several other types of substructures bound by lipid membranes known as *organelles*. Although the beautiful multicoloured rings that fringe some hot springs are composed of archaea, and bacteria can also form visible colonies, individual living organisms that are of dimensions visible to humans are invariably eukaryotic and multicellular, often containing complex and highly organized assemblages of cells. An adult human, for example, has been estimated to contain greater than 10^13^ cells [2].

**Figure 1.**
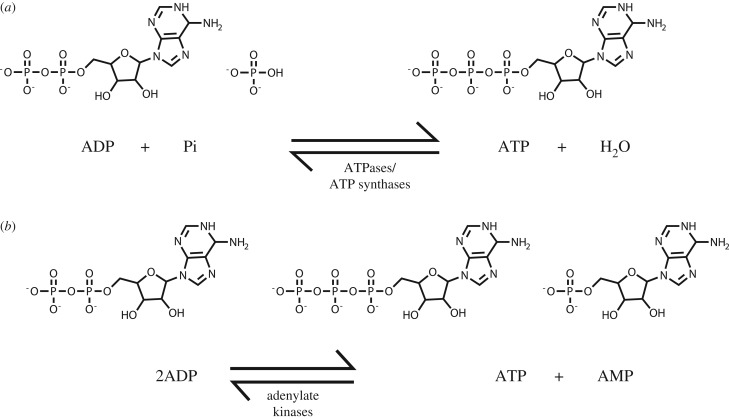
Reactions catalysed by (*a*) ATPases or ATP synthases and (*b*) adenylate kinases.

All living cells require a constant supply of energy to grow and reproduce, to repair wear and tear, to maintain intracellular compositions often very different from their extracellular environment and to perform other specialized functions such as secretion of materials into their surroundings, electrical communication or movement. They obtain this energy in one of three ways: (i) by *heterotrophy*, as practised by animals, in which organisms and their component cells ingest or absorb reduced organic compounds and oxidize them, usually to CO_2_, by the process known as *catabolism* (hence ‘*keeping the home fires burning*'); (ii) by *photoautotrophy* as in green plants, in which the organism uses energy from sunlight to fix and convert CO_2_ into reduced organic compounds, and (iii) by *lithoautotrophy* in which the organism oxidizes reduced compounds of mineral origin. Despite these radically different lifestyles, all cells conserve some of the energy released in these processes using a common currency, by converting the nucleotide adenosine diphosphate (ADP) and phosphate ions (Pi) into adenosine triphosphate (ATP) (figure 1*a*). Under normal conditions, the equilibrium for the reaction that interconverts ATP and ADP + Pi lies well in favour of ATP hydrolysis (i.e. towards the left-hand side of figure 1*a*). However, by coupling ATP synthesis to oxidation of reduced compounds, or to photosynthesis, most cells maintain ATP concentrations around 10-fold higher than those of ADP, which is many orders of magnitude away from the equilibrium position of the hydrolysis reaction. A useful analogy can be drawn between ATP and ADP and the chemicals in a rechargeable battery: catabolism or photosynthesis ‘charge up the battery' by converting ADP to ATP, while the majority of other cellular functions require energy and are mostly driven (directly or indirectly) by being coupled to the conversion of ATP back to ADP. Clearly, to sustain life this ‘battery' needs to remain fully charged (ATP : ADP ≈ 10 : 1), which requires that the rate of ATP production by catabolism and/or photosynthesis is exactly balanced by the rate at which ATP is consumed by energy-requiring processes, including the biosynthesis of macromolecules required for cell growth (*anabolism*). There is no *a priori* reason why these two complex processes should automatically remain in balance, and the fact that they usually do is because cells contain sensitive control systems to preserve that balance. One of the most important of these is the *AMP-activated protein kinase* or *AMPK*, which forms the main topic of this article.

**Figure 2.**
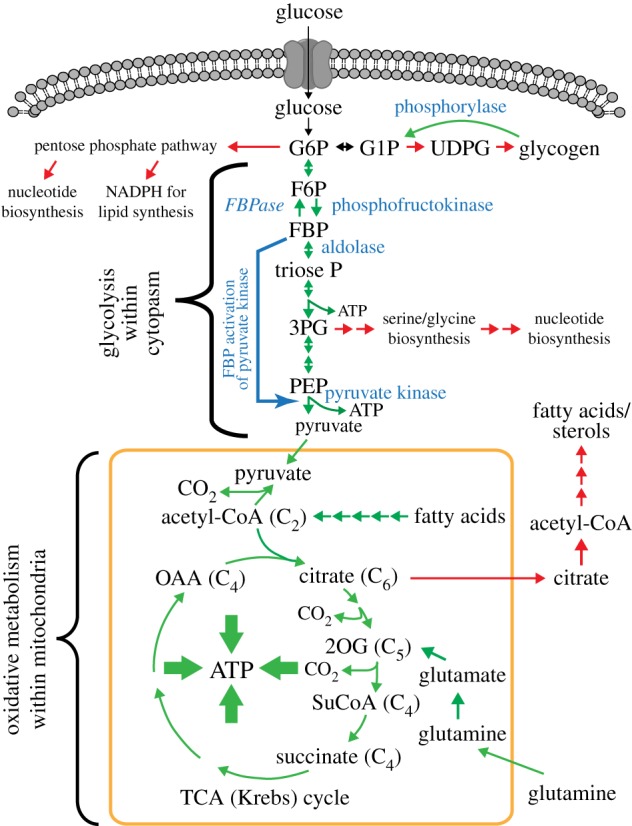
Schematic of the major catabolic pathways (and selected anabolic pathways) in a typical animal cell, showing how they are distributed between the cytoplasm and the mitochondria. Key to intermediates: G6P, glucose-6-phosphate; G1P, glucose-1-phosphate; UDPG, UDP-glucose; F6P, fructose-6-phosphate; FBP, fructose-1,6-bisphosphate; triose-P, dihydroxyacetone phosphate or glyceraldehyde-3-phosphate; 3PG, 3-phosphoglycerate; 2PG, 2-phosphoglycerate, PEP, phosphoenolpyruvate; 2OG, 2-oxoglutarate; OAA, oxaloacetate. (Online version in colour.)

While discussing ATP and ADP, I should also mention the third adenine nucleotide, adenosine monophosphate or AMP, which plays an important role in this story. Although produced in a few other reactions, the major cellular source of AMP appears to be the reaction catalysed by *adenylate kinases*, enzymes that catalyse the interconversion of the three adenine nucleotides (figure 1*b*). There is little change in free energy in this reaction, so it is readily reversible with an equilibrium constant close to 1 such that, at equilibrium, [ATP].[AMP]/[ADP]^2^ will be ≈1. If this reaction is indeed at or near equilibrium (which appears to be the case in most eukaryotic cells), and cells maintain an ATP : ADP ratio of 10 : 1 as discussed above, it follows that the ratio of ATP : AMP will be 100 : 1. In a cell with a fully charged ‘battery', the concentration of AMP will, therefore, be 100-fold lower than that of ATP and 10-fold lower than that of ADP. However, if the ADP : ATP ratio starts to rise (i.e. the ‘battery is going flat'), and the adenylate kinase reaction is at equilibrium, then it is easy to show [3] that the AMP : ATP ratio will rise as the square of the ADP : ATP ratio, making the former a much more sensitive indicator of falling cellular energy status than the latter. This was realized by Sir Hans Krebs as long ago as 1964, who in his Croonian lecture to the Royal Society stated: ‘it is noteworthy that the tissue concentrations of AMP are subject to much greater percentage changes under physiological conditions than the concentrations of ATP or ADP, or the ratio ATP/ADP' [4]. These observations help to explain why the AMPK system, a sensor of cellular energy status, responds primarily to changes in AMP : ATP ratio rather than those in ADP : ATP, as will be discussed in more detail later. There are also at least three metabolic enzymes that respond to cellular energy status independently of AMPK (figure 2): (i) *phosphofructokinase* (involved in *glycolysis*, the initial pathway of glucose breakdown); (ii) *phosphorylase* (involved in the breakdown of glycogen, the storage form of glucose), and (iii) *fructose-1,6-bisphosphatase* (*FBPase*), which reverses the phosphofructokinase step, albeit using a different reaction, during de novo synthesis of glucose (*gluconeogenesis*) in the liver. *Phosphofructokinase* and *phosphorylase* are catabolic enzymes that are activated by decreased cellular energy status, while *FBPase* is an anabolic enzyme that is inhibited by decreased cellular energy status. It is interesting that, like AMPK, all three of these energy-sensing metabolic enzymes respond primarily to AMP and ATP, rather than to ADP and ATP [5–7].

## 2. In animal cells, most ATP is generated in mitochondria

Some of the central metabolic pathways of animal cells are summarized in figure 2. The initial conversion of glucose to pyruvate, called *glycolysis*, occurs in the main compartment of the cell called the *cytoplasm*, whereas oxidative metabolism, which uses molecular oxygen to bring about complete oxidation of pyruvate (as well as other reduced carbon compounds such as fatty acids) to CO_2_, occurs exclusively in the *organelles* called *mitochondria*. Glycolysis is capable of generating ATP rapidly, but is inefficient in the sense that only a small amount of ATP is produced for each molecule of glucose consumed (just two molecules of ATP per molecule of glucose, as opposed to greater than 30 by complete oxidation to CO_2_).

**Figure 3.**
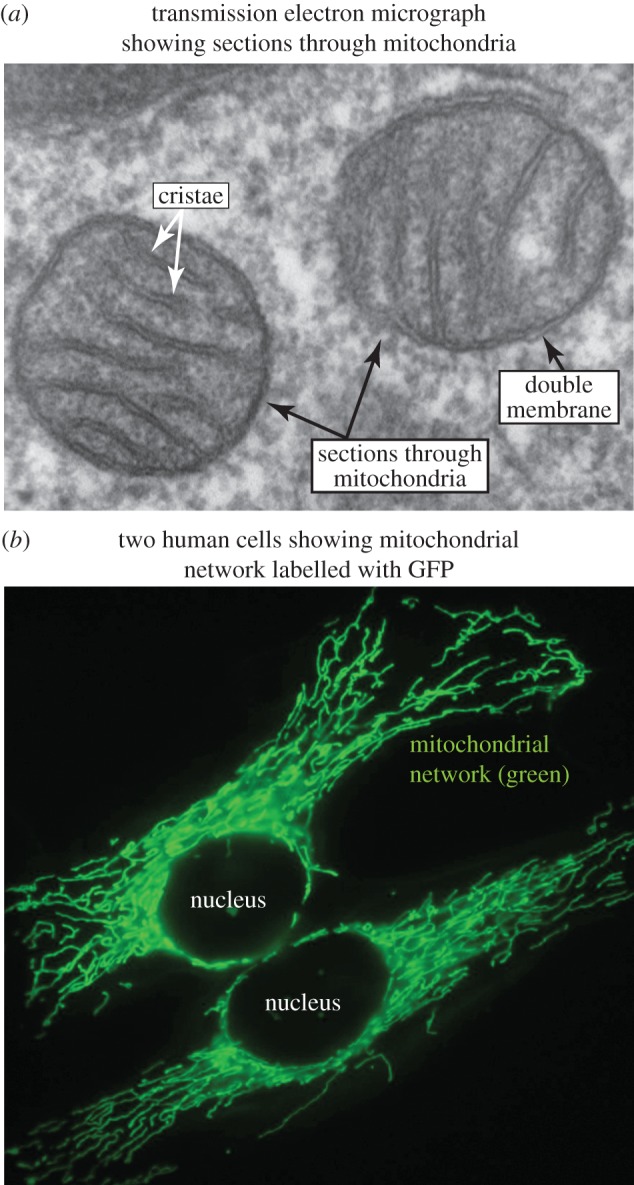
(*a*) Transmission electron micrograph and (*b*) fluorescence micrograph of cells showing mitochondria. The image of a human lung cell in (*a*) (placed in the public domain by Louisa Howard) reveals the *double membrane* surrounding mitochondria, and the invaginations of the inner membrane known as *cristae*. The fluorescence micrograph in (*b*) (by Simon Troeder, CC BY 4.0), which is at much lower magnification, shows mitochondria in two human cells expressing a mitochondrially targeted green fluorescent protein, and reveals that mitochondria actually form a branching network of tubules. The image in (*a*) is of two tubules sampled as thin sections, which therefore appear to be spherical or ovoid. (Online version in colour.)

The critical event that led to the development of eukaryotic cells (including eventually human cells) is now believed to have been an *endosymbiosis*, i.e. a mutually beneficial engulfment by an *archaeal* host cell of aerobic (oxygen-using) *bacteria* [8]. The *archaeal* host may have evolved at a time when little oxygen was available in the atmosphere, and was most likely only capable of limited catabolism of glucose by glycolysis. By the time of the hypothetical endosymbiotic event, the concentration of oxygen in the atmosphere had almost certainly built up to much higher levels due to the actions of photosynthetic bacteria, and aerobic bacteria had by then mastered the complex (but energetically rewarding) task of using molecular oxygen to completely oxidize reduced carbon compounds to CO_2_. There was, therefore, a major potential advantage for the host cell to nurture and retain the bacterial invaders, rather than attempting to expel or destroy them as might normally occur with a parasite. As the host and endosymbiont may not initially have been able to exchange ATP and ADP (which is now carried out by membrane transporters of the *ADP/ATP carrier* (*AAC*) family [9]), it has been argued that the early advantage to the host may simply have resided in the ability of the endosymbiont to reduce the intracellular concentration of oxygen, whose rising levels might otherwise have been becoming toxic [10]). However, once ATP and ADP could be exchanged across the membrane of the endosymbiont, the benefit to the host cell would have been greatly enhanced. In return, the enveloped bacterium would have found a much richer source of carbon nutrients than would be the case outside of the host cell. So mutually advantageous was this happy arrangement that it is thought that the bacteria quite quickly became completely integrated into the host cell, and became what we now call mitochondria (figure 3). Although this endosymbiotic origin of mitochondria was controversial when first proposed by Lynn Margulis (formerly Lynn Sagan) in 1967 [8], the evidence in its favour is now rather overwhelming. For example, the analysis of amino acid sequences shows that many mitochondrial proteins are closely related to their bacterial counterparts, whereas cytoplasmic proteins tend to be more imilar to their archaeal relatives [11]. In addition, mitochondria still contain their own DNA that replicates independently of the DNA in the nucleus, as well as machinery for synthesizing the RNAs and proteins encoded by that DNA. Despite this, the majority of the genes that were acquired from the bacterium during the endosymbiotic event have, for reasons that are not completely clear, been transferred into nuclear DNA, so that mitochondria are now quite incapable of living freely on their own. Note that despite the oval ‘bacterium-like' appearance of mitochondria in transmission electron micrographs (figure 3*a*), this is a rather misleading impression caused by the cutting of thin sections prior to electron microscopy. Mitochondria often occur in cells as elongated and even branched tubular networks (figure 3*b*), as well as in more fragmented, vesicular forms; these alternate forms exist in a dynamic equilibrium mediated by fission and fusion, and which form predominates is dependent upon conditions.

By a similar (although probably much later) endosymbiotic process, photosynthetic eukaryotes such as green plants are thought to have arisen when a host eukaryotic cell (which probably already contained mitochondria) engulfed blue-green algae, a type of photoautotropic bacterium, which then became chloroplasts. This relationship between blue-green algae and chloroplasts is more obvious than that between mitochondria and aerobic bacteria, and consequently was proposed much earlier, by the Russian biologist Constantin Mereschkowsky in 1905 [12]. This event allowed plants to convert ADP to ATP using energy from sunlight, and to fix and reduce carbon dioxide to generate glucose that could then be stored in the form of the polysaccharide starch, still one of the major sources of food for humans. Plant cells also contain mitochondria, and when the light fades at night they break down starch stored during the day into glucose, to fuel ATP production by their mitochondria. Cells in the subterranean parts of the plant (the roots) use mitochondria for ATP production even during the day.

Like the bacteria and blue-green algae from which they are thought to derive, mitochondria (figure 3*a*) and chloroplasts are surrounded by double membranes. Embedded in their inner membranes are chains of redox-active proteins and lipids (called the *respiratory chain* in mitochondria, and *photosystems* in chloroplasts). These transfer electrons down energy gradients, with the electrons that have passed down the mitochondrial respiratory chain eventually being used to reduce oxygen and H^+^ ions (protons) to water. Some of the energy released during electron transfer down these chains is conserved by pumping protons from the inner compartments of the mitochondrion (or chloroplast) to the outside. This creates a gradient of electrical charge, as well as a chemical gradient of protons across the inner membrane, representing yet another store of energy. Although these gradients can be used to directly drive some energy-requiring processes in the organelles themselves, they are mainly dissipated by remarkable multi-subunit protein machines known as ATP synthases or F_1_F_o_ ATPases [13]. These contain F_o_ sub-complexes embedded in the inner membrane, which rotate within the membrane as protons pass back through them, requiring 10–15 protons to pass for every 360° rotation. This rotation is then transmitted via a central stalk to the F_1_ sub-complex located on the inside of the inner membrane, which contains the ATP synthase enzyme that generates three molecules of ATP from ADP and Pi for every rotation (the system can also operate in reverse, using energy from ATP hydrolysis to pump protons out). As already mentioned, mitochondria can generate around 36 molecules of ATP per molecule of glucose by this process, as opposed to the two generated by the more limited pathway of glycolysis in the cytoplasm. Mitochondria and chloroplasts can also export ATP into the cytoplasm by means of *ATP/ADP carriers* that exchange ADP for ATP across their inner membranes. Mitochondria, therefore, generate the majority of the ATP in most animal cells.

A defining feature of mitochondria is that their inner membranes are greatly invaginated, containing infoldings called *cristae* (figure 3*a*). This greatly increases the surface area of membrane that can be occupied by the respiratory chains or photosystems, as well as by the ATP synthases, thus enhancing the production of ATP per unit volume. It has been argued [14] that the acquisition of mitochondria in the early eukaryote may have allowed the large increase in cell size that subsequently occurred, because it meant that ATP could now be generated throughout the volume of the cell, rather than just close to the plasma membrane where it occurs in bacterial or archaeal cells, perhaps placing a limit on the ultimate size of the latter. This innovation may also have allowed the proliferation of other membrane-bound organelles, such as the *nucleus*, *endoplasmic reticulum*, *Golgi apparatus*, *endosomes* and *lysosomes*, as well as processes involving membrane traffic such as *exocytosis* and *endocytosis* (fusion of intracellular lipid vesicles with the plasma membrane, thus delivering materials to the exterior and vice versa). These processes, as well as others such as bodily movement by skeletal muscles in animals, and the electrical communication that is required to control it, are particularly expensive in terms of energy turnover, and none of them are found in bacteria. Such innovations may ultimately have allowed the development of more complex multicellular organisms such as humans, which may not have been possible using archaeal or bacterial cells because they have to use energy with such great efficiency.

When the endosymbiotic engulfment of aerobic bacteria first occurred to generate what we now know as mitochondria, one can envisage that the host cell would have needed to develop a system that could monitor the primary output of the endosymbiont, i.e. ATP, and signal back for it to step ATP production up or down according to the demand. The AMPK, the main topic of this review, monitors the energy status of cells by sensing the relative levels of AMP, ADP and ATP, as discussed below. When it is switched on as cellular energy status falls, one of the things it does is to promote the production of new mitochondria, as well as assisting with the disposal of old, damaged regions of the mitochondrial network and recycling their contents (see §6.2). I would, therefore, argue that, following the endosymbiosis event that created mitochondria, AMPK may have evolved to provide the critical regulatory link between the new endosymbiont and its host.

## 3. Protein kinases and phosphatases act as molecular switches

**Figure 4.**
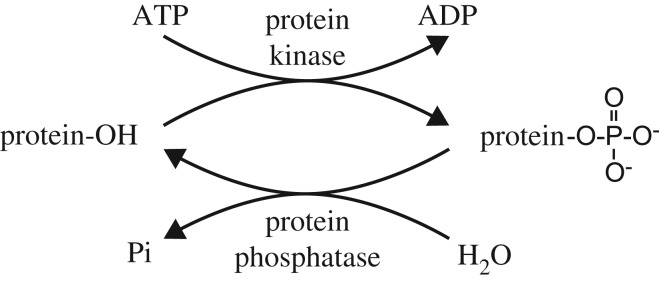
The distinct reactions catalysed by protein kinases and protein phosphatases.

As its name suggests, AMPK is a *protein kinase*, an enzyme that catalyses *protein phosphorylation*, the transfer of the terminal phosphate group of ATP onto an amino acid side chain of a *target protein* carrying a hydroxyl group, thus creating a *phosphate ester* (figure 4). Of the 20 commonly occurring amino acids that make up proteins, only serine, threonine or tyrosine carry hydroxyl groups, and individual protein kinases tend to be specific either for the aliphatic side chains of serine or threonine (like AMPK), or for the aromatic side chain of tyrosine. Protein phosphorylation is reversed by enzymes called *protein phosphatases*, which catalyse the hydrolysis of the phosphate ester to regenerate the unmodified target protein. Note that protein kinases and phosphatases catalyse distinct reactions (figure 4), each of which are essentially irreversible under cellular conditions (the high ATP : ADP ratio in cells drives kinase reactions to completion, while the high concentration of cellular water drives protein phosphatase reactions to completion). This means that a kinase–phosphatase cycle acts as a *molecular switch*, reversibly switching the target protein between the dephosphorylated and phosphorylated states according to the relative catalytic activities of the kinase and phosphatase. Creation of a phosphate ester adds two negative charges to an amino acid side chain that was previously uncharged (figure 4), and this can have profound effects on the function of the target protein. For example, if the target protein is an enzyme it can switch its catalytic activity on or off; in other cases, it may trigger a protein : protein interaction that subsequently alters the subcellular location of the protein or targets it for degradation. Cells also contain other molecular switch mechanisms, including GTP-binding proteins (*G proteins*), which are switched between GDP-bound and GTP-bound forms by upstream modifying proteins called *guanine-nucleotide exchange factors* (GEFs) and *GTPase-activator proteins* (GAPs). Protein kinase : phosphatase cycles and G protein GEF : GAP cycles can, therefore, act as molecular switches (although only acting as truly digital, ‘on–off' switches under certain conditions [15,16]) and are thus analogous to the transistors in an electronic circuit. Indeed, the analogy can be taken further, because there are greater than 500 protein kinases, greater than 100 protein phosphatases, and greater than 100 G proteins encoded in the human genome (not counting regulatory subunits). Because protein kinase/phosphatase pairs often regulate the activity of other protein kinases and phosphatases, with the help of G proteins and additional molecular switch mechanisms they form complex networks of signalling pathways within the cell, analogous to the central processor unit (CPU) of a computer. Just as in a computer, these signalling networks receive *input information* via receptors and sensor proteins (one example of which is AMPK itself). The signalling network then processes the information to convert it into appropriate *output,* which takes the form of changes in either the function or the quantity of downstream target proteins. The ultimate downstream targets include enzymes and other proteins involved in metabolism, cell growth and division, movement, secretion and so on.

## 4. Structure, occurrence and regulation of AMPK

### 4.1. AMPK is expressed in almost all eukaryotes

AMPK exists universally as complexes of three different protein subunits (*heterotrimers*) comprising a catalytic *α* subunit that carries the protein kinase activity, and regulatory *β* and *γ* subunits. Each of these subunits has a characteristic pattern of amino acids and, even though random mutations have meant that the sequences have diverged between different species, computer algorithms are available that allow these patterns to be recognized. The development of DNA sequencing technology means that we now have almost complete genomic DNA sequences for many different organisms, and one can confidently state that, with some interesting exceptions discussed in §4.2, all eukaryotic genomes, whether from protozoa, fungi, plants or animals, contain genes encoding the *α*, *β* and *γ* subunits of AMPK. This fits with the proposal, mentioned in §2, that AMPK evolved relatively soon after the endosymbiotic event that led to the development of eukaryotes.

### 4.2. Eukaryotes that lack AMPK: intracellular parasites

There are a small number of eukaryotes that lack genes encoding AMPK subunits. One is the microsporidian parasite *Encephalitozoon cuniculi*, which is derived from fungi and can infect mammalian cells, including cells of immunocompromised humans with AIDS. *E. cuniculi* has the smallest known genome of any eukaryote, encoding only 29 conventional protein kinases (as opposed to greater than 500 in humans) and lacks genes encoding AMPK subunits [17]. It is an obligate intracellular parasite and has no free-living form other than metabolically inert spores. Although its genome does encode glycolytic enzymes, it lacks functional mitochondria-producing ATP, although there is good evidence that it has lost these rather than never having had them [18,19]. Intriguingly, *E. cuniculi* has genes encoding ATP/ADP carriers, which in other eukaryotes are used to export ATP from mitochondria and chloroplasts in exchange for ADP, but in *E. cuniculi* these proteins are located in the outer cell membrane [20]. Thus, this organism may be able to survive without AMPK, because it ‘steals’ ATP from its eukaryotic host cell, which will contain AMPK to maintain energy balance on behalf of the parasite.

Other examples of eukaryotes lacking AMPK [21] are also parasites that primarily live inside other eukaryotic cells. They include *Plasmodium falciparum* and *P. berghei,* the causative agents of malaria in humans and rodents, respectively. Interestingly, two closely related species, *P. gallinaceum* and *P. relictum*, which cause malaria in birds, do have genes encoding AMPK subunits [22], which suggests that the human and rodent parasites have lost genes encoding AMPK relatively recently. Like *E. cuniculi*, malaria parasites spend a large part of their life cycle reproducing inside other eukaryotic cells (red blood cells), and may have been able to dispense with AMPK, because their host cells provide it anyway. Thus, these parasites can be regarded as the exceptions that ‘prove the rule' that AMPK is universal within eukaryotic cells.

### 4.3. Multiple forms of AMPK arose by whole genome duplication

During the evolution of the *vertebrates* (animals with backbones), it is now thought that two successive rounds of duplication of the whole genome occurred [23]. Evidence in favour of this hypothesis includes the genome sequence of the marine chordate Amphioxus (*Branchiostoma floridae*), which is believed to have diverged from the vertebrate lineage just prior to these events, and where most genes are present as single copies [24]. Two whole genome duplications would initially have led to there being four copies of every gene, but because most of these would have been redundant in function, in most cases three were lost by subsequent mutations, and their function is now often encoded by a single gene once again. However, about 20–30% of genes in the human genome have retained two, three or even four copies as a vestige of these gene duplication events. Such closely related genes within a single genome are known as *paralogues* or, if it is clear that they have arisen from the two rounds of whole genome duplication that occurred during vertebrate development, as *2R-ohnologues* (honouring Susumu Ohno, the Japanese scientist who first proposed the whole genome duplication hypothesis [23]). Intriguingly, the 20–30% of genes that have retained 2R-ohnologues are highly enriched in proteins involved in cell signalling and regulation [25,26]. Once formed, the 2R-ohnologues could have accumulated mutations and slowly diverged in sequence, regulation and tissue distribution, and it has been argued that this allowed variations in regulatory behaviour to have appeared in different tissues expressing different 2R-ohnologues. This increased diversity could then have contributed to the development of the complex variety of tissues and organs that exist in vertebrates.

The AMPK heterotrimer is a good example of this principle, because in humans there are two genes encoding the *α* subunit (generating *α*1 and *α*2 isoforms), two encoding the *β* subunit (generating *β*1 and *β*2 isoforms) and three encoding the *γ* subunit (generating *γ*1, *γ*2 and *γ*3 isoforms). There is strong circumstantial evidence that these are all 2R-ohnologues [27]. The seven genes can combine to form up to 12 heterotrimeric combinations, which show variations in their tissue distribution, regulatory properties and subcellular locations [27,28].

### 4.4. Mechanism of energy-sensing by AMPK

**Figure 5.**
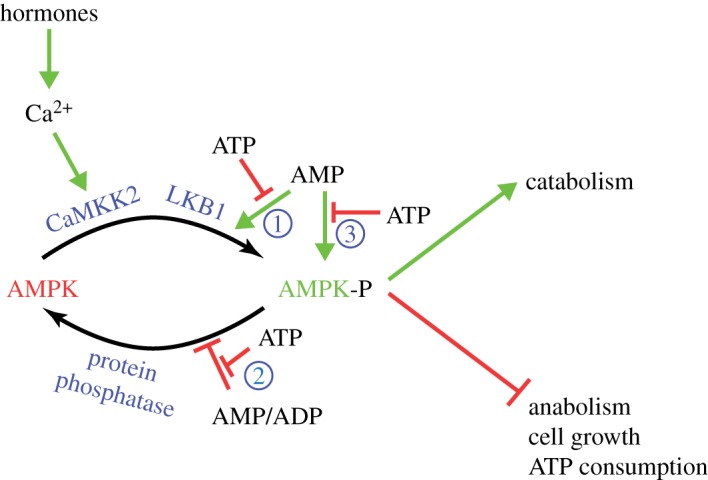
Tripartite mechanism for activation of AMPK by increases in the cellular AMP : ATP ratio, and by the non-canonical Ca^2+^-CaMKK pathway. Binding of AMP to the AMPK-γ subunit causes activation by (1) promoting phosphorylation by LKB1; (2) inhibiting dephosphorylation by protein phosphatases and (3) allosteric activation. Binding of ADP at higher concentration can mimic effect (2), whereas biding of ATP antagonizes all three effects. Increases in intracellular Ca^2+^ activate CaMKK2, which phosphorylates the same site on AMPK (Thr172) as LKB1. (Online version in colour.)

As mentioned in §3, protein kinases often phosphorylate and activate other protein kinases, forming chains of protein kinases known as *protein kinase cascades*. AMPK is no exception to this, and is only significantly active after it has been phosphorylated at a specific threonine residue within the region of the *α* subunit that carries the protein kinase activity, usually referred to as Thr172 due to its position in the sequences of the *α*1 and *α*2 subunits from the rat [29]. This is catalysed by two distinct upstream kinases: LKB1 [30–32] and CaMKK2 [33–35], potentially enabling AMPK to assimilate input information from two converging upstream signalling pathways. Thus, CaMKK2 is activated by increases in intracellular Ca^2+^ ion concentrations, which occurs in response to many hormones acting on cells (figure 5, see §4.8). By contrast, LKB1 appears to be constantly active, but forms a key part of the mechanism by which AMPK is able to sense the energy status of the cell, which will now be described.

As discussed in §1, cells normally maintain their ATP : ADP ratio at about 10 : 1, representing a fully charged ‘battery'. If the rate at which ATP is consumed by energy-requiring processes exceeds the rate at which it can be regenerated by catabolism or photosynthesis, the ADP : ATP ratio will rise. It might be thought that a system that monitored the cellular energy state would sense the ADP : ATP ratio but, as discussed in §1, changes in AMP : ATP ratios are generally much larger than those in ADP : ATP. It is, therefore, interesting that AMPK primarily monitors AMP : ATP, although it does also appear to be able to detect changes in ADP : ATP. AMP binding causes activation of AMPK by three complementary mechanisms, all of which are antagonized by ATP (figure 5): (1) increasing the rate at which LKB1 phosphorylates Thr172; (2) decreasing the rate at which protein phosphatases dephosphorylate Thr172 and (3) increasing the activity of AMPK already phosphorylated on Thr172 (*allosteric activation*). This tripartite activation mechanism makes the system very sensitive to small changes in the cellular AMP : ATP ratio [36]. Note that all three effects appear to be due to the binding of AMP to AMPK itself, rather than to the upstream kinase or phosphatase. As net Thr172 phosphorylation (the summation of mechanisms (1) and (2)) increases the kinase activity of AMPK by greater than 100-fold, while allosteric activation (mechanism (3)) increases the kinase activity by a further 10-fold, AMP binding can theoretically increase the activity by greater than 1000-fold overall. In practice, however, AMPK activity in living cells under physiological conditions probably normally varies over quite a narrow range [37]. Because binding of ADP to AMPK can mimic (albeit only at almost 10-fold higher concentrations than AMP [37]) mechanisms (1) and (2), but not (3), increases in ADP : ATP ratio would also be detected.

### 4.5. Structure of the AMPK heterotrimer

**Figure 6.**
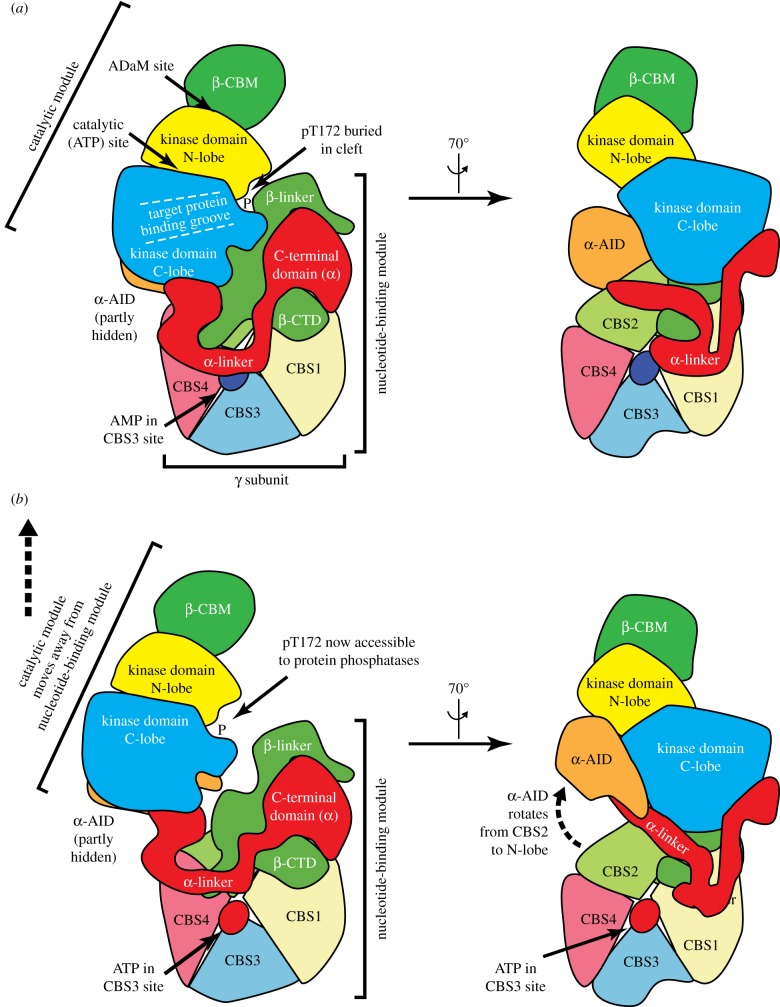
Schematic of changes in domain disposition in the AMPK heterotrimer when AMP (*a*) rather than ATP (*b*) is bound to the CBS3 site on the *γ* subunit. The model at the top is based on the structure of the human *α*1*β*2*γ*1 complex, crystallized with Thr172 phosphorylated in the presence of AMP and cyclodextrin (PDB file 4RER) [39]; the two views are rotated by 70° with respect to each other around the y axis. The model at the bottom (of the same two views) is a hypothetical structure in which the catalytic and nucleotide-binding modules have shifted apart upon displacement of AMP by ATP, with consequent release of the α-linker from the CBS3 site.

Structures for three almost complete AMPK heterotrimers, i.e. *α*1*β*1*γ*1, *α*1*β*2*γ*1 and *α*2*β*1*γ*1, have now been obtained through analysis of X-ray diffraction by crystals. The three structures are quite similar; all have AMP bound and with Thr172 in the phosphorylated state [38–40]. They point to the fact that AMPK is a remarkable molecular machine for converting changes in adenine nucleotide ratios into changes in protein kinase activity. Usually, different functions within proteins are carried out by discretely folded regions called *domains*. Figure 6*a* shows two schematic views (derived from crystal structures) of the approximate domain dispositions of one AMPK heterotrimer; the two views being rotated 70° around the vertical axis with respect to each other. The kinase activity is carried by the *kinase domain* at the *N-terminal end* of the *α* subunit; as in other protein kinases, the kinase domain consists of a small N-terminal lobe (N-lobe, yellow in figure 6) and a larger C-terminal lobe (C-lobe, in blue). One of the substrates for the kinase activity, ATP, binds (as an Mg·ATP^2−^ complex) in the *catalytic site*, a deep cleft between these two lobes, while the other, the target protein that is to be phosphorylated, binds partly in a groove on the surface of the C-lobe (the *target protein-binding groove*, figure 6*a*, left), and partly in the cleft formed between the N- and C-lobes when they close around ATP (see §6.1 below for more details of this binding site). Phosphorylation of Thr172 by upstream kinases causes a local rearrangement that completes the binding site for the target protein. This binding mode brings the serine or threonine residue of the target protein adjacent to the terminal phosphate group of ATP, ready for transfer [41].

The kinase domain on the AMPK-α subunit is followed by an auto-inhibitory domain (AID, orange in figure 6) that, in other partial structures, binds to the N- and C-lobes of the kinase domain (see figure 6*b*, right), clamping them in a *conformation* (a specific folded state) that is much less active in catalysis. However, in the complete structures with the activator AMP bound, the AID has rotated away from the N-lobe and interacts between the C-lobe and the *γ* subunit instead (figure 6*a*, right), with the kinase domain now in an active conformation.

Following the AID on the *α* subunit is a region of flexible polypeptide called the α-linker, which connects the AID to the C-terminal domain (in red in figure 6). The latter interacts with the C-terminal domain of the *β* subunit (β-CTD, green), which in turn interacts with the *γ* subunit, thus forming the core of the heterotrimeric complex. The *β* subunit also contains a carbohydrate-binding module (β-CBM, the green domain at the top of the views in figure 6) that causes a portion of AMPK to bind to glycogen particles in cells. The function of this remains unclear, although it has been speculated that AMPK may be able to sense the status of stores of glycogen, which is the major cellular reserve of glucose in animal cells [42].

Turning now to the *γ* subunits, when their amino acid sequences were first determined, it was realized that they contained patterns of amino acid sequence, termed CBS motifs, that are repeated four times, one after the other [43]. These four tandem repeats (CBS1–CBS4) come together in a pseudo-symmetrical manner to form the four quadrants of a disc-shaped structure, generating four clefts in the centre with a narrow aqueous tunnel between them (figure 5). These clefts form the binding sites for the regulatory adenine nucleotides AMP, ADP and ATP, although only three are actually used. The critical site appears to be that formed by CBS3. Consistent with this, the α-linker binds to the surface of the *γ* subunit containing the CBS3 site when AMP is bound (figure 6*a*). The α-linker appears to play an important role in the changes in shape that activate the kinase, as discussed in the next section.

### 4.6. Conformational changes upon AMP binding to AMPK

Unfortunately, there are no complete crystal structures with ATP bound at the CBS3 site, so we do not know the exact changes in *conformation* (shape) of the complex that occur when ATP replaces AMP at that site. However, there is evidence from small angle X-ray scattering in solution [44], and from luminescence energy transfer between probes located on the *α* and *γ* subunits [39], that the heterotrimer adopts a more compact conformation on AMP binding, and conversely that the *α* and *γ* subunits move apart when ATP is bound. The α-linker can be envisaged as a flexible hinge connecting two rather distinct regions of the heterotrimer: (i) the *catalytic module*, containing the β-CBM and the kinase domain and AID of the *α* subunit, and (ii) the *nucleotide-binding module*, containing the *γ* subunit and the C-terminal domains of the *α* and *β* subunits (figure 6). The displacement of the α-linker from the CBS3 site when ATP binds is thought to allow these two modules to move apart [45], thus causing two effects: (i) it allows the AID to rotate back into its inhibitory position behind the kinase domain; (ii) it exposes the phosphate group on Thr172, which in the AMP-bound structure is buried in a narrow cleft between the two modules, to dephosphorylation by protein phosphatases. This proposed change in conformation on binding of ATP rather than AMP at the CBS3 site, as depicted rather speculatively in the bottom panel of figure 6, would therefore explain mechanisms (2) and (3) (see §4.4) by which AMP binding causes activation of AMPK.

If CBS3 is the critical site where the activator AMP binds in competition with the inhibitor ATP, What is the purpose of the other two (CBS1 and CBS4)? Although we do not have a complete answer, what is clear is that the phosphate groups of the three bound nucleotides come close together in the aqueous tunnel in the centre of the *γ* subunit, where some amino acid side chains bind phosphate groups of the nucleotide in more than one site. The three sites thus interact, and it has been suggested that the presence of adenine nucleotides in the other two sites alters the shape of the CBS3 site such that it binds AMP with almost 10-fold higher affinity than ATP [46]. This explains how the *γ* subunit is able to sense small changes in AMP even in the presence of 100-fold higher concentrations of ATP, which could potentially be envisaged as a difficult task given the chemical similarities of the two nucleotides (figure 1*b*).

### 4.7. The ADaM site, salicylate and aspirin

The cleft between the β-CBM and the kinase domain N-lobe forms the binding site for another interesting class of AMPK activator. Most of these are synthetic compounds developed in high-throughput screens by pharmaceutical companies (see §6.4). However, many people believe that these drugs are mimicking a naturally occurring metabolite that binds in the same site, which is why it is referred to as the Allosteric Drug and Metabolite (ADaM) site (figure 6*a*, left) [47]. One natural product that does bind to the ADaM site and activates AMPK, although it only occurs naturally in plants, is *salicylate* [48]. Salicylate is a hormone released by regions of plants that have been infected by pathogens, which triggers a defence response in uninfected regions [49]. The bark of white willow (*Salix alba*), from which the name salicylate is derived, is a rich source of salicin, a glucosyl derivative of salicylate. Extracts of willow bark were used in the eighteenth century by the Reverend Edward Stone to treat himself and his parishioners of ‘agues' (fevers), resulting in a paper read before the Royal Society in 1763 [50]. He was, in fact, rediscovering a remedy that had been known in ancient times, and that was described in Sumerian stone tablets and Egyptian papyrus documents dating from the 3rd millennium BCE [51]. Salicin was also successfully tested for the treatment of rheumatic fever by Dr Thomas Maclagan at the Royal Infirmary in my home town (Dundee) in the 1870s [52], and because he left some patients untreated this is sometimes regarded as one of the first controlled clinical trials. However, the use of salicylates really took off when Bayer introduced the synthetic derivative acetyl salicylate (aspirin) in 1899 [51]. In 1971, Sir John Vane discovered that aspirin irreversibly inhibited cyclo-oxygenase, the key enzyme of biosynthesis of lipid mediators such as prostaglandins and thromboxane, with the latter promoting platelet aggregation and hence blood clotting [53]. However, once it enters the bloodstream, aspirin is rapidly broken down to salicylate with a half-life of just a few minutes, while the resulting salicylate is stable for many hours [54]. Moreover, while the anti-blood-clotting effects of aspirin are undoubtedly mediated by the inhibition of thromboxane synthesis, it remains uncertain whether inhibition of cyclo-oxygenase explains all of the other effects of aspirin. Indeed, it has been suggested that some of its anti-inflammatory [55] and even pain-relieving effects [56] might be mediated instead by AMPK activation.

### 4.8. The CaMKK2 pathway and effects of hormones on AMPK

Like LKB1, CaMKK2 can activate AMPK by phosphorylating Thr172 on the AMPK-α subunit [33–35], and it is itself activated by interaction with the Ca^2+^-bound form of the small Ca^2+^-binding protein *calmodulin*. Many hormones, which act via receptors that activate G proteins or protein-tyrosine kinases, cause release from the cell membrane of the second messenger, inositol trisphosphate, which in turn releases Ca^2+^ from intracellular storage sites. Such hormones, therefore, activate AMPK via the CaMKK2 pathway. This includes two hormones or mediators that act on the *endothelial cells* that line blood vessels [57], i.e. *thrombin* and *vascular endothelial cell growth factor* (*VEGF*) [58,59]. Thrombin is produced from its inactive precursor *prothrombin* as one of the final steps in the blood-clotting cascade, whereas VEGF is released by cells that are hypoxic (short of oxygen) and promotes the growth of new blood vessels (*angiogenesis*). Indeed, there is evidence that the *α*1 isoform of AMPK is required for angiogenesis induced by VEGF [59].

The Ca^2+^-CaMKK2 pathway for activation of AMPK is also involved in the response of *neurons* (nerve cells) that trigger sensations of hunger and hence promote feeding behaviour; these are located in the hypothalamus, the small brain region that links the nervous system and the endocrine (hormonal) system. Hormones that trigger activation of AMPK via CaMKK2 in the hypothalamus include ghrelin [60,61], which is released from the stomach during fasting. It is thought to act on neurons immediately upstream of other neurons that express *agouti-related protein* (*AGRP*), which trigger feeding behaviour when they are excited. There is also evidence that AMPK activation in these upstream neurons triggers a positive feedback mechanism that ensures that they remain active even after ghrelin release has ceased. If this is correct, AMPK in these neurons will only be switched off when ‘satiety' signals act upon them; these are most likely to be opioid peptides, released in response to ‘satiety' hormones such as leptin [60] from other neurons that express the opioid precursor protein, *pro-opiomelanocortin* (*POMC*). Consistent with the role of CaMKK2 in the response to ghrelin, acute inhibition of CaMKK2 by direct injection of an inhibitor into the hypothalamus of normal mice led to reduced food intake and weight loss, while mice with a genetic knockout of CaMKK2 consumed less and were resistant to weight gain induced by an energy-rich diet [62]. As discussed in §5, the ancestral role of AMPK may have been in the sensing of nutrients, especially glucose. It is, therefore, intriguing that this ancient nutrient-sensing system has been co-opted in mammals to control a complex behavioural response, i.e. feeding. While AMPK in the hypothalamus appears to be mainly controlled by hormones such as ghrelin and leptin released from elsewhere in the body, some neurons in the hypothalamus also respond directly to deficiency of glucose in an AMPK-dependent manner [63]. I discuss the mechanism of glucose-sensing by AMPK in other cell types in the next section.

## 5. Sensing of glucose by AMPK

### 5.1. Glucose-sensing may be an ancient role of the AMPK system

It has been known for many years that the version of AMPK found in brewer's or baker's yeast (a fungus that can be grown in a unicellular form) is required for responses to glucose starvation [64], a stress that activates the kinase [65] by phosphorylation of the threonine residue equivalent to Th172 [66]. Evidence that the version of AMPK found in plants also responds to starvation for carbohydrate came from studies of the moss *Physcomitrella patens*; plants deficient in the genes encoding AMPK grew normally if the light was kept on constantly, but failed to grow when the plants were maintained in a more physiologically relevant 12 L : 12 D cycle [67]. Green plants make hexose sugars from CO_2_ in their chloroplasts when the light is switched on, so a period of darkness is the equivalent of starvation for glucose. Taken together, these findings suggest that the response to glucose starvation may have been an ancient function of AMPK in the common evolutionary ancestor of fungi, plants and animals.

In mammals, the sensing of cellular energy status, by the mechanisms described in §§ 4.4–4.6, has always been regarded as the classical or ‘canonical' role of AMPK. Although it has been known for many years that starving cells of glucose activates AMPK [68], it was generally assumed that this was because this manipulation reduced the production of ATP by glucose catabolism, and thus increased cellular AMP and ADP. However, it has recently become clear that AMPK can sense glucose by a non-canonical mechanism that does not necessarily involve changes in cellular adenine nucleotide concentrations. In mouse cells grown in culture, reducing the concentration of glucose in the medium caused an activation of AMPK that correlated with increased phosphorylation of Thr172, which was not accompanied by any increases in AMP : ATP or ADP : ATP ratios as long as an alternative carbon source was still present [69]. A similar process also occurred under more physiological conditions in living animals, since overnight starvation of mice, which caused blood glucose to drop from 9 to 3 mM, was associated with increased Thr172 phosphorylation of AMPK within the liver, without any changes in adenine nucleotides. In other cell types, including some human cells in culture, there were small increases in AMP : ATP and ADP : ATP ratios when glucose was removed from the medium, possibly because these cells are more dependent upon the more glucose-hungry glycolytic pathway for their ATP production. Nevertheless, glucose starvation still activated AMPK in these cells, albeit to a smaller extent, when they expressed an AMPK mutant in which binding of AMP at the critical CBS3 site on the *γ* subunit was ablated, thus preventing the response to agents that increase intracellular AMP [70]. Thus, even in cells where removal of glucose from the medium does cause some decrease in energy status, there is an AMP/ADP-independent component to the mechanism by which glucose lack is sensed.

### 5.2. Mechanism of glucose-sensing by AMPK

How does mammalian AMPK sense glucose, assuming that it is not by sensing changes in AMP, ADP and ATP? One initial clue came from findings that the *β*1 and *β*2 subunits of AMPK are modified at their N-termini by covalent modification using the C14 fatty acid, myristic acid [71,72]. This modification, *N-myristoylation*, occurs in other proteins and often causes the modified protein to attach to membranes, with the hydrophobic myristoyl group being buried in one layer of the double layer of phospholipids (the *phospholipid bilayer*) in all membranes. Myristoylation of the *β*1 subunit was found to be necessary for increased Thr172 phosphorylation in response to glucose deprivation, and also for AMPK to cluster at an unidentified membrane location near the nucleus in response to glucose deprivation [72].

The next clue came from studies of the protein Axin, a scaffold protein previously known for its role in another signalling pathway (*Wnt signalling*). Knockdown of Axin expression caused accumulation in mouse liver of fatty acids in the form of triacylglycerols, especially after overnight starvation [73]. This was reminiscent of what might be expected for reduced activation of AMPK, because when AMPK is activated it reduces fat storage by inhibiting fat synthesis and promoting fat oxidation (see §6.2). AMPK activation after overnight starvation was indeed reduced by Axin knockdown. It was subsequently shown that Axin forms a binary complex with the upstream kinase for AMPK, LKB1, and that AMP then promotes the formation of a ternary complex involving Axin, LKB1 and AMPK. Axin is, therefore, an *adapter protein* that promotes the phosphorylation and activation of AMPK by LKB1 by bringing the upstream and downstream kinases together in a complex. This effect was promoted by AMP, thus explaining mechanism (1) of the tripartite mechanism for activation of AMPK by AMP, already discussed in §4.4.

The missing link between membrane association, Axin and glucose-sensing was identified from a screen that searched for other proteins interacting with Axin, which yielded the protein p18 (also known as Lamtor1) [74]. p18/Lamtor1 is a resident protein of *lysosomes*, another organelle that is specific to eukaryotes, which occur in plant and fungal cells as larger structures called *vacuoles*. The interior of lysosomes or vacuoles is maintained at a lower pH than that of the cytoplasm due to the pumping of protons into the interior by a rotary pump driven by ATP hydrolysis (the *vacuolar ATPase* or *v-ATPase*), which is related to the F_1_F_o_ ATPase that synthesizes ATP in the mitochondria (see §2). Lysosomes contain degradative enzymes that are active at this low pH and that break down proteins, lipids and polysaccharides. The latter are delivered to lysosomes by membrane trafficking events such as *autophagy* (which delivers intracellular materials) and *endocytosis* (which delivers extracellular materials), thus enabling recycling of their components. p18/Lamtor1 is a component of the pentameric *Ragulator* complex, which is resident on the lysosome partly because of N-terminal modifications of p18/Lamtor1 by the C14 and C16 fatty acids myristic and palmitic acid [75] (recall that the *β* subunits of AMPK are also subject to N-terminal myristoylation), and partly because it interacts with the v-ATPase [76].

Further analysis of these interactions showed that, upon starving cells for glucose, a multiprotein complex involving the v-ATPase, Ragulator, LKB1 and AMPK was formed on the cytoplasmic surface of lysosomes; because each component of this complex itself contains multiple subunits (at least 14, 5, 3 and 3, respectively), we refer to this as a *super-complex*, or more specifically as the *Axin-based AMPK activation complex*.

### 5.3. What is being sensed and what is the sensor?

These findings still did not reveal how glucose was being sensed. One key question was whether it was glucose itself that was recognized, or a product of its metabolism. By systematically knocking down expression of enzymes of glycolysis and the pentose phosphate pathway (two of the major pathways for the initial metabolism of glucose, figure 2), it became clear that repression of AMPK activation by glucose required its metabolism at least through the first three steps of glycolysis, i.e. as far as fructose-1,6-bisphosphate (FBP). That it was FBP itself that was being sensed came from experiments showing that of all ten intermediates of the glycolytic pathway, only FBP caused dissociation of the Axin-based AMPK activation complex present in a subcellular fraction containing lysosomal membranes, isolated from glucose-starved cells.

If FBP is being sensed, what is the sensor? One possibility was that aldolase, the enzyme that metabolizes FBP in the glycolytic pathway (figure 2), might be ‘moon-lighting' as a sensor. Consistent with this idea, knockdown of aldolase expression led to AMPK activation even in cells incubated in high glucose [69]. However, as knockdown would affect not only the putative glucose-sensing role of aldolase but also its metabolic role, alternative approaches were required. More convincing results came from studies with a previously described mutant of aldolase (D34S), which still binds FBP with normal affinity even though the maximal catalytic rate (*k*_cat_) is reduced by 3–4 orders of magnitude [77]. This mutant would, therefore, be expected to have FBP bound even when the overall flux of glycolysis was low due to lack of glucose. Consistent with the model, AMPK was no longer activated by glucose starvation in cells expressing this mutant [69]. These results suggest that it is aldolase that is *unoccupied by FBP* that signals reduced availability of glucose, and that activates AMPK.

**Figure 7.**
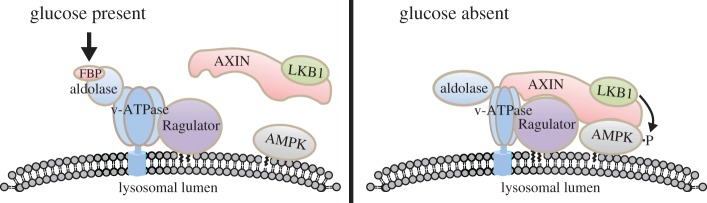
Model for the non-canonical, AMP-independent activation of AMPK upon glucose starvation. In the presence of glucose, a high flux through glycolysis means that aldolase, which associates with the v-ATPase on the lysosomal membrane, has fructose-1,6-bisphosphate (FBP) bound, preventing interaction of the Axin : LKB1 complex, which is cytoplasmic, with the v-ATPase and the Ragulator. AMPK may already be, at least partly, located on the lysosome due to N-myristoylation of the *β* subunit. On removal of glucose, aldolase is now largely unoccupied by FBP and a conformational change allows the Axin : LKB1 complex to bind to the v-ATPase and the Ragulator. LKB1 now phosphorylates and activates AMPK: whether this causes dissociation of the active AMPK from the lysosomal membrane remains unclear at present. Diagram courtesy of Chensong Zhang and Shengcai Lin, based on [69]. (Online version in colour.)

Intriguingly, aldolase had previously been shown to interact with the lysosomal v-ATPase in both yeast and mammalian cells, and even to be required for the correct assembly of the v-ATPase complex [78–80]. Even more interestingly, the association between aldolase and FBP in yeast was reported to increase dramatically when glucose was present in the medium, which led to the proposal that aldolase was a sensor of glucose availability, albeit at that time only thought to be involved in the regulation of the v-ATPase [79]. Taken together, these findings led to the model shown in figure 7. When glucose is available and the flux through glycolysis is, therefore, high, aldolase that is bound to the v-ATPase will have FBP bound to it, and this prevents the binding of the Axin : LKB1 complex to the v-ATPase : Ragulator complex. Meanwhile, AMPK may already be partly associated with the lysosomal membrane due to the N-myristoylation of its *β* subunit. On removing glucose from the medium, aldolase now becomes unoccupied by FBP, and this causes a conformational change that is transmitted to the v-ATPase, causing the Axin : LKB1 complex to bind to the v-ATPase : Ragulator complex and to recruit AMPK into the super-complex. This, in turn, leads to the phosphorylation and activation of AMPK by LKB1. We presume that the activated AMPK then dissociates from the lysosome to perform many of its downstream functions, although that has not yet been directly demonstrated.

### 5.4. Why fructose-1,6-bisphosphate, why aldolase and why lysosomes?

When glucose enters cells, there is a choice between its metabolism by glycolysis, which in most cells is primarily a catabolic pathway that generates ATP, or by anabolic pathways such as the pentose phosphate pathway or glycogen synthesis (figure 2). FBP is the product of the enzyme *phosphofructokinase*, which catalyses the first irreversible step in the glycolysis pathway. Once FBP has been reached, its fate is, therefore, largely committed to catabolism. This makes FBP a good signal that there is sufficient glucose available for catabolic purposes, so that it is not necessary for AMPK to switch on alternative catabolic pathways.

Note that this is not the only use of FBP as a signal, because some isoforms of pyruvate kinase, which catalyse the final step in glycolysis (figure 2), are subject to *allosteric activation* by FBP. Some flux downstream of aldolase can, in fact, be used for anabolic rather than catabolic purposes, because 3-phosphoglycerate (3PG) is the starting point for the synthesis of the amino acid serine and glycine, which are required not only for protein synthesis but also for nucleotide synthesis (figure 2). Activation of pyruvate kinase by FBP would ensure that when glucose is readily available, most of the flux would go through glycolysis to pyruvate, thus generating ATP. However, if glucose (and hence FBP) were in short supply, a larger proportion of the flux might be diverted into the serine/glycine biosynthesis pathway.

Aldolase may be a good sensor to indicate the availability of FBP, because the maximum concentration of FBP in cells incubated with high glucose is around 10 µM [69], but higher concentrations are required to saturate binding of FBP to aldolase. Thus, as FBP concentration varies across its physiological range, the amount bound to aldolase will increase proportionately.

One can only speculate as to why glucose-sensing by AMPK should take place on the lysosome, but one interesting point is that many present day protists, such as amoebae, feed primarily by *phagocytosis*, i.e. the engulfment of extracellular particles by pinching off of membrane-bound vesicles (*phagosomes*) into the cytoplasm, which are then delivered to lysosomes for digestion of their contents. All eukaryotic cells also recycle their own surplus contents by *autophagy*, in which regions of the cytoplasm, including organelles, are engulfed within membrane vesicles (*autophagosomes*) that are then delivered to lysosomes for digestion (see §6.2). The lysosome can, therefore, be viewed as the ‘gut' or digestive system of the single cell, where nutrients such as glucose, fatty acids and amino acids are generated by digestion of polysaccharides, lipids and proteins. It is, therefore, seems logical that these nutrients might be sensed there.

### 5.5. Interplay between the AMPK and target-of-rapamycin pathways

One intriguing facet of the recent findings of a role for the Ragulator complex and lysosomal membranes in glucose-sensing by AMPK is the close links with the *mechanistic target-of-rapamycin complex-1* (*mTORC1*) *pathway*, another ancient nutrient-sensing pathway that appears to be present in almost all eukaryotes. *Rapamycin* is an antibiotic originally isolated from a soil bacterium collected on the remote Pacific outcrop of Easter Island, which is known to the locals as Rapa Nui [81]. The target-of-rapamycin (TOR) was discovered as two related genes in brewer's yeast in which loss-of-function mutations prevented the growth inhibitory effects of rapamycin [82]. These genes were sequenced in 1993 and shown the following year to be closely related to a mammalian protein (originally called RAFT1, now mTOR) that binds to a complex between rapamycin and a cellular binding protein called FKBP12 [83].

mTOR, which in humans is encoded by a single gene, is now known to exist as two multi-subunit complexes, i.e. mTORC1 containing mTOR and Raptor, and mTORC2 containing mTOR and Rictor, with both complexes also containing additional components. Although closely related to kinases that attach phosphate groups to lipids, mTORC1 and mTORC2 are, in fact, protein kinases. In most respects, mTORC1 acts in the opposite direction to AMPK, in that it is activated by the availability of nutrients, especially amino acids, and by phosphorylating downstream targets distinct from AMPK promotes anabolic pathways, especially the synthesis of proteins required for rapid cell growth [84]. It is, therefore, intriguing that the Ragulator complex, which is involved in the activation of AMPK by glucose starvation, is also involved in the activation of mTORC1 by amino acids. When stimulated by amino acids, by mechanisms that are still being elucidated [84], the Ragulator complex acts as a guanine-nucleotide exchange factor (GEF) for RagA or RagB, converting them to their active GTP-bound forms. RagA/B are alternative components of a heterodimeric G protein that also contains RagC or RagD; when RagA/B have bound GTP, and RagC/D have bound GDP, mTORC1 is recruited to the lysosome via binding of Raptor to the Rag heterodimer [85]. However, to be fully active mTORC1 requires another lysosomal G protein, *Rheb*, to be in its GTP-bound form. This occurs when the *TSC complex*, which carries a GTPase-activator protein (GAP) activity for Rheb, has dissociated from the lysosome following its phosphorylation by protein kinases acting downstream of growth factors [86]. Thus, mTORC1 is analogous to an electronic *coincidence circuit* in that it requires two conditions to be met before it becomes active: (i) amino acids are available; (ii) growth factors are present. A third condition for mTORC1 to be active, and thus for cell growth to be permitted, is that AMPK should be inactive, because AMPK inactivates mTORC1 by dual mechanisms: (i) it phosphorylates the TSC complex at distinct sites that oppose the effects of the protein kinases downstream of growth factors, thus inactivating Rheb [87]; (ii) it phosphorylates Raptor at two sites [88]. Although in both cases, this leads to inactivation of mTORC1 in intact cells, in neither case are the effects of phosphorylation well understood at the molecular level.

## 6. Downstream effects of AMPK, and its potential as a drug target

### 6.1. Recognition of downstream targets by AMPK

**Figure 8.**
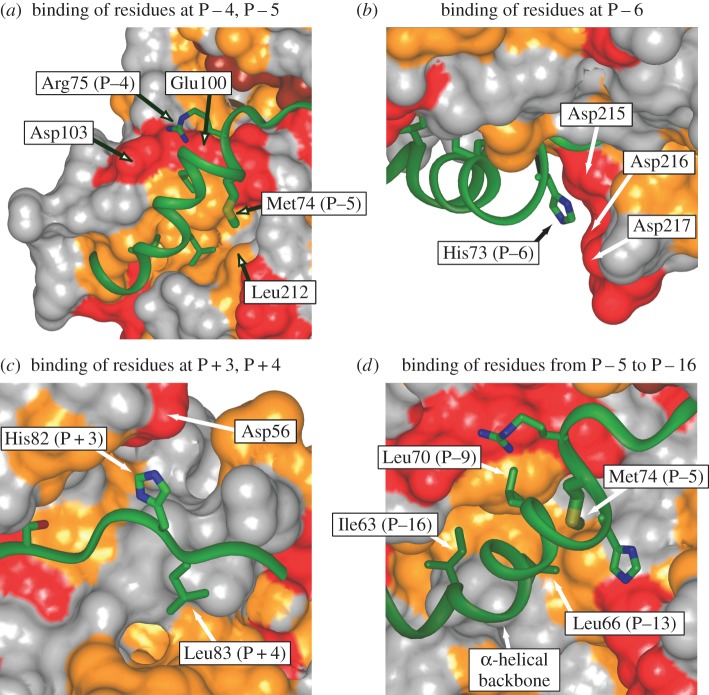
AMPK recognizes its targets by means of complementary interactions between amino acid side chains surrounding the target serine/threonine residues and side chains from the kinase domain of AMPK. The images were based on a model of the kinase domain with the sequence around Ser79 on the downstream target ACC bound to it [41]. The kinase domain is in ‘spacefilling' representation, while the ACC sequence is in ‘cartoon' representation with the polypeptide backbone represented as a narrow green ribbon, and specific side chains shown with carbon atoms in green, nitrogen in blue and sulfur in orange. (*a*) The basic side chain (Arg75) at P − 4 (i.e. four residues N-terminal to the target serine) binds to an acidic patch (red) formed by Asp103 and Glu100 from the kinase domain, while the hydrophobic side chain at P − 5 (Met74) interacts with a hydrophobic pocket (orange) containing Leu212 from the kinase domain; (*b*) the basic side chain (His73) at P − 6 interacts with an acidic patch (red) formed by Asp215, Asp216 and Asp217; (*c*) the basic side chain at P + 3 (His82) interacts with Asp56 from the kinase domain, while the hydrophobic side chains at P + 4 (Leu 83) interacts with a hydrophobic pocket (orange) on the surface of the α-KD; (*d*) the α-helix running from P − 16 to P − 5 binds in a hydrophobic groove (the target protein-binding groove, figure 6), with the hydrophobic side chains of Ile63 (P − 16), Leu66 (P − 13), Leu70 (P − 9) and Met74 (P − 5) binding in the groove. (Online version in colour.)

Although the majority of protein kinases, like AMPK, are specific for serine or threonine residues, the average protein will contain tens or even hundreds of these, while usually only one or two, or often none at all, are phosphorylated on a particular protein. Like some other protein kinases, AMPK achieves the remarkable feat of recognizing a few hundred specific serine and threonine residues, out of perhaps up to a million in a typical cell, because it recognizes the pattern of surrounding amino acids. AMPK has been shown to recognize sequences of the type β-Φ-(β,X)-X-X-S/T-X-X-X-Φ, where *β* is an amino acid with a basic, positively charged side chain (arginine, lysine or histidine), *Φ* is an amino acid with a bulky hydrophobic side chain (methionine, leucine, isoleucine, phenylalanine or valine), X is any amino acid, S/T is the serine or threonine phosphorylated and (*β*, X) indicated that the basic side chain (*β*) can be at either of the two positions [41,88,89]. This *sequence motif* is recognized by complementary amino acids in the catalytic region of AMPK, which was established by modelling the binding of the sequence around serine-79 on the classical downstream target, acetyl-CoA carboxylase (ACC1), to the kinase domain of AMPK (figure 8) [41]. These complementary amino acids are mostly not contiguous within the amino acid sequence of AMPK, but are brought together by the way it folds up. Although the structures shown in figure 8 were the result of modelling (based on the structures of closely related kinases) rather than actual structural analysis, the model was confirmed by mutating individual residues involved in binding, both on the kinase and on the target [41].

The development of phosphorylation sites for a particular kinase such as AMPK on many different proteins is a good example of *convergent evolution*, in which the selection of random mutations causes sequences of proteins that are not closely related by their ancestry to become more similar, at least over short stretches. The AMPK recognition motif occurs with too high a frequency in protein sequences predicted by the human genome to be particularly useful in identifying new target proteins, but where a target protein has been identified by other means, it has been very useful in identifying the exact site(s) of phosphorylation.

### 6.2. AMPK activation causes a metabolic switch from anabolism to catabolism

**Figure 9.**
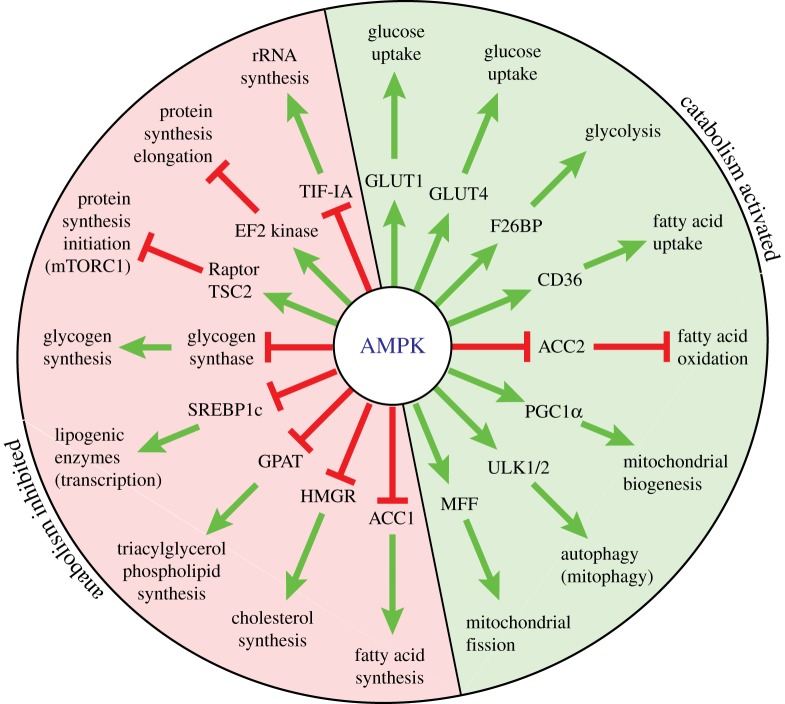
Summary of catabolic pathways (green) switched on and anabolic pathways (red) switched off when AMPK is activated. Green arrows indicate activation and red lines with bars across the end indicate inhibition. Key to acronyms: GLUT1/GLUT4, glucose transporter-1/-4; F26BP, fructose-2,6-bisphosphate; CD36, cluster of differentiation 36, a fatty acid transporter; ACC1/ACC2, acetyl-CoA carboxylase-1/-2; PGC1*α*, peroxisome proliferator-activated receptor co-activator-1*α*; ULK1/2, UNC-51-like kinase-1/-2; MFF, mitochondrial fission factor; HMGR, HMG-CoA reductase; GPAT, glycerol phosphate acyltransferase; SREBP1c, sterol response element-binding protein-1c; TSC2, tuberous sclerosis complex protein-2; EF2, elongation factor-2; TIF-IA, transcription initiation factor-IA. (Online version in colour.)

We now know of at least 60 target proteins for AMPK, most of which are phosphorylated on just one or two serine/threonine residues [90]; it is anticipated that the list will eventually run into hundreds. Many of the early downstream targets to be discovered were metabolic enzymes: in general, AMPK switches on catabolic enzymes involved in the generation of ATP, while switching off anabolic enzymes involved in the ATP-consuming processes of synthesis and storage of macromolecules (figure 9). Thus, while slowing cell growth AMPK at the same time helps to restore energy balance in cells in which energy status has been compromised.

As AMPK is activated by the depletion of glucose as well as cellular energy, it makes sense for it to accelerate the use of any remaining glucose by enhancing its uptake into cells by the glucose transporters GLUT1 [91] and GLUT4 [92]. At the same time, AMPK promotes the initial metabolism of glucose (glycolysis) in some cell types by phosphorylating and activating the PFKFB2 and PFKFB3 isoforms of the enzyme that converts fructose-6-phosphate to *fructose-2,6-bisphosphate* (F26BP), an allosteric activator of the key phosphofructokinase step in glycolysis [93,94]. However, as glucose runs low, it also makes sense for cells to switch to the use of alternative fuels such as fatty acids. Thus, AMPK promotes uptake of fatty acids into cells via the fatty acid transporter CD36 [95], while also phosphorylating and inactivating ACC that synthesizes malonyl-CoA, an inhibitor of fatty acid uptake into mitochondria [96]. Thus, AMPK activation promotes ATP synthesis by increasing both uptake and oxidation of fatty acids. As well as these rapid effects, in the longer term AMPK promotes fatty acid oxidation by enhancing the expression of oxidative enzymes within mitochondria, including enzymes that catalyse the Krebs or TCA cycle (the prominent cycle of reactions within mitochondria shown in figure 2, first defined by Hans Krebs) [97]. AMPK also enhances *mitochondrial biogenesis* [98], i.e. the synthesis of new mitochondrial components, by activating or stabilizing *PGC-1α* [99,100], a transcriptional activator that enhances expression of many mitochondrial proteins, as well as the replication of mitochondrial DNA [101]. This ensures that AMPK promotes ATP production not only from fatty acids and any pyruvate derived from glycolysis but also from amino acids such as glutamine, which feeds into the Krebs cycle by the pathway of *glutaminolysis* (figure 2). Glutamine is generated by breakdown of not only dietary proteins but also muscle proteins during periods of prolonged starvation, and is usually the most abundant amino acid in the bloodstream.

A final catabolic action of AMPK, brought about by phosphorylation of the protein kinases ULK1/ULK2 [102], is to promote *autophagy* (literally *self-eating*), a process in which cells engulf within intracellular membranes some of their own cytoplasmic proteins and organelles that are surplus to requirements. The resulting membrane-bound vesicles (*autophagosomes*) deliver their contents to *lysosomes*, where they are degraded and their components (such as glucose and amino acids) recycled, either for catabolism or for reuse. In cells starved of nutrients, activation of autophagy by AMPK increases the chances of survival via this form of self-cannibalism. On the other hand, damaged segments of mitochondria are also engulfed and degraded by the special form of autophagy known as *mitophagy,* even in unstarved cells. The use of molecular oxygen for mitochondrial metabolism comes at a price, in that by-products known as *reactive oxygen species*, especially superoxide (^•^O_2_^−^), hydrogen peroxide (H_2_O_2_) and hydroxyl free radicals (^•^OH), are generated. Mitochondrial proteins, lipids and DNA are particularly susceptible to damage by these highly reactive chemicals*.* Remarkably, cells seem to be able to segregate off damaged regions of mitochondria by the process of *mitochondrial fission*, in which segments of mitochondrial tubules are pinched off [103]. These are then delivered to autophagosomes so that their components can be recycled. AMPK activation, which occurs in cells that are short of either glucose or ATP, promotes not only mitochondrial fission by phosphorylating *mitochondrial fission factor* (*MFF*) [104] but also *mitophagy* itself [102]. Consistent with this, cells lacking AMPK tend to accumulate abnormal or dysfunctional mitochondria [105,106]. Thus, AMPK helps to maintain the entire life cycle of mitochondria, not only promoting the production of new mitochondrial components (mitochondrial biogenesis) but also by disposing of old, damaged mitochondria by mitochondrial fission and mitophagy. This is consistent with the idea, proposed in §2, that the AMPK system evolved as a key interface between the host cell and its bacterial endosymbiotic partners.

In addition to these catabolic processes that are switched on by AMPK, it switches off most ATP-consuming, anabolic pathways (figure 9). It was originally discovered for its ability to phosphorylate and inactivate ACC and 3-hydroxy-3-methyl-Coenzyme A reductase (HMGR) [107–109]. Besides producing malonyl-CoA that inhibits fatty acid oxidation (see above), ACC is the ATP-requiring enzyme that produces malonyl-CoA used in the synthesis of fatty acids. These tasks may be partly divided between two closely related isoforms of acetyl-CoA carboxylase, ACC1 and ACC2, both of which are inactivated by AMPK; ACC2 has been reported to be associated with mitochondria [110], and may produce the pool of malonyl-CoA that regulates fatty acid oxidation. On the other hand, HMG-CoA reductase is the key regulatory enzyme involved in the synthesis of cholesterol and other isoprenoids. AMPK has also been reported to inactivate glycerol phosphate acyl transferase (GPAT), an enzyme involved in triacylglycerol and phospholipid synthesis [111]. In addition to these rapid, direct effects to inhibit lipid synthesis, AMPK also appears to repress expression of lipogenic enzymes, in part, by phosphorylation of the transcription factor SREBP1c [112]. Other direct targets of AMPK involved in anabolism include the muscle [113] and liver [114] isoforms of glycogen synthase, which are phosphorylated at equivalent N-terminal residues to cause inactivation of the enzyme; the need to co-localize AMPK with glycogen synthase may be part of the rationale underlying the presence of a carbohydrate-binding module on the AMPK-β subunits (see §4.5). Finally, it has been estimated that 20% of total oxygen uptake (a measure of energy usage) in rapidly proliferating cells is required for the synthesis of proteins, and 15% for the synthesis of nucleic acids (DNA and RNA) [115]. As already discussed in §5.5, AMPK inactivates mTORC1 via phosphorylation of TSC2 and Raptor, thus inhibiting the initiation of synthesis of many proteins, especially those required for rapid cell growth [116]. In addition, AMPK acutely inhibits protein synthesis by phosphorylating and activating *elongation factor-2* (*EF2*) *kinase*, a kinase that inhibits the elongation phase of protein synthesis by phosphorylating EF2 [117]. This last mechanism would ensure that protein synthesis pauses whenever cells run low on energy. Turning now to nucleic acid synthesis, the most abundant cellular nucleic acid is ribosomal RNA (rRNA), which constitutes up to 80% of the RNA in most cells; ribosomes are the structures that synthesize new proteins and are mainly composed of rRNA. Indeed, AMPK inhibits rRNA synthesis via direct phosphorylation of the key transcription factor, TIF-IA [118]. AMPK, therefore, restrains the synthesis of all of the major components of growing cells, i.e. lipids, proteins and nucleic acids, thus restraining cell growth.

### 6.3. Non-metabolic targets of AMPK

Although AMPK was at one time mainly thought to regulate metabolism, it is becoming increasingly clear that it regulates many other energy-requiring processes, one example being cell division. The cell division cycle is conventionally divided into four phases: (i) *G1 phase*, a first phase of cell growth; (ii) *S phase*, when cellular DNA is duplicated; (iii) *G2 phase*, a second phase of cell growth, and (iv) *M phase* or *mitosis*, when the duplicated DNA is distributed equally between the two daughter cells. Once activated above a certain threshold, AMPK arrests progress through the cell cycle in G1 phase, in part, by increasing the expression of inhibitory proteins that block the action of protein kinases required for progression into S phase [119,120]. AMPK thus indirectly inhibits the synthesis of DNA by blocking entry into S phase, adding to its more direct effect on the synthesis of rRNA described in the last section.

Owing to limitations on space, it is not possible to discuss all of the known targets of AMPK. However, another process that is very expensive in energy usage is the firing of *action potentials*, the electrical impulses that carry messages along the long processes called axons that emanate from the cell bodies of nerve cells (*neurons*). Remarkably, the constant rate of ATP turnover in some regions of the brain can be comparable with that in leg muscles during running of a marathon, and it has been estimated that the firing of action potentials accounts for between 25% and 50% of this energy expenditure, with the transmission of chemical signals between neurons (a downstream consequence of the firing of action potentials) accounting for all bar 15% of the remainder [121]. Having a large brain in humans is, therefore, very expensive in terms of energy consumption, requiring constant ingestion of large quantities of food to maintain it. Among the major consumers of energy in neurons (and indeed other cells) are the Na^+^/K^+^-ATPases, membrane transporters that pump these ions across membranes against concentration gradients, driven directly by ATP hydrolysis. The Na^+^/K^+^-ATPase transports 3 Na^+^ ions out of cells in exchange for 2 K^+^ ions that enter, thus creating concentration gradients of Na^+^ and K^+^ ions (high outside and high inside, respectively), as well as a charge gradient (negative inside). These transporters, therefore, create a large driving force for the re-entry of Na^+^ ions into the cell, and a smaller driving force for the exit of K^+^ ions. A local depolarization of the membrane (when the charge gradient becomes less negative or even positive) causes firing of *action potentials* by triggering the opening of *voltage-gated Na^+^ channels.* These channels are selective for Na^+^ ions and open when they sense a depolarization of the membrane; their presence is one of the key characteristics of *excitable cells* such as neurons. Opening of Na^+^ channels triggers more depolarization and thus creates a positive feedback, initiating a kind of chain reaction in which the opening of voltage-gated Na^+^ channels in neighbouring areas of membrane generates a wave of depolarization that is propagated down the axon, carrying a message. At the site where the action potential was initially triggered, the depolarization is quite rapidly reversed by the closure of the Na^+^ channels and the opening of voltage-gated K^+^ channels, which rapidly restore the original polarity by allowing K^+^ ions to flow in an outward direction. Some rapidly opening K^+^ channels are responsible for terminating individual action potentials, while others (e.g. the delayed rectified K^+^ channel, Kv2.1) open and close more slowly and are responsible for increasing the resting membrane potential, reducing the propensity of voltage-gated Na^+^ channels to open again and thus start another action potential. Interestingly, Kv2.1 is a target for AMPK, which phosphorylates its cytoplasmic, C-terminal tail, causing channel opening to shift to more negative membrane potentials [122]. Thus, the channel will open earlier upon membrane depolarization, restraining the firing of more action potentials. In elegant confirmation of this model, micro-injection of active AMPK into cultured rat neurons reduced the frequency of action potentials induced by a current pulse, whereas an inactive mutant had no effect [122]. By this mechanism, AMPK has the potential to conserve energy in the central nervous system by reducing the rate of firing of action potentials, and may thus enhance the survival of neurons that are under energetic stress. Putting this rather flippantly, it could be argued that it is better for neurons to work slowly than not to work at all!

### 6.4. Uses of AMPK activators and inhibitors to treat human disease

Based on the ability of AMPK to switch cell metabolism from an anabolic mode (in which macromolecules are synthesized and stored) to a catabolic mode (in which stored nutrients are broken down and their components oxidized to CO_2_), it was suggested in 1999 [123] that AMPK activation by small molecule drugs might have benefits in the treatment of metabolic disorders such as obesity and Type 2 diabetes. Type 2 diabetes is a disorder characterized by a high blood glucose that, unlike the Type 1 form, is not initially caused by lack of *insulin* (a hormone that promotes glucose uptake by muscle and inhibits glucose production by the liver) but by a failure of cells to properly respond to insulin, the phenomenon known as *insulin resistance*. Although the causes of insulin resistance remain poorly understood, it is strongly associated with obesity, and particularly appears to occur when organs such as the liver and muscle store excessive quantities of nutrients, especially fats.

This prediction that AMPK activation could be used to treat Type 2 diabetes [123] was rapidly supported by findings that *metformin*, the primary drug currently used to treat the disorder, activated AMPK in intact cells and *in vivo* [124]. Metformin was derived from an old herbal remedy, i.e. the plant *Galega officinalis* or Goat's Rue, which was said by John Parkinson (court physician to James I of England) to be ‘effectual against all infections’ [125]. Metformin and a related drug, *phenformin*, are synthetic *biguanide* derivatives of galegine (the active ingredient of Goat's Rue, which proved to be too toxic for use in humans and presumably also for goats!) and were introduced for the treatment of Type 2 diabetes in the 1950s. Phenformin was subsequently withdrawn, because in a small number of cases it caused the life-threatening side-effect of *lactic acidosis*. However, this complication is much less frequent with metformin, which has become the front-line drug treatment in Type 2 diabetes. Being derived from a herbal remedy, the actual direct targets of metformin and phenformin were unknown until two groups reported in 2000 that they were inhibitors of Complex I of the mitochondrial respiratory chain [126,127] (incidentally, this explains the risk of lactic acidosis as the production of lactic acid, an end-product of glycolysis, would occur when inhibition of the respiratory chain became severe). As these drugs would inhibit mitochondrial ATP production, they would also activate AMPK, which was indeed demonstrated [124]. That this was due to increases in cellular AMP was confirmed by showing that metformin did not activate AMPK in cells that expressed a mutation in the *γ* subunit that prevented binding of AMP [70]. Intriguingly, at least fifty natural products of plants, many of which have been used in traditional Chinese medicine, have now been shown to activate AMPK [128]. Although the mechanism of action of the majority of these remains unknown, several, including berberine [129], arctigenin [130], galegine [70] and phloretin [131], inhibit Complex I of the respiratory chain, like metformin and phenformin. Most of these natural products are so-called secondary metabolites of plants, i.e. are not required for plant growth and development under optimal conditions. However, like galegine, many are toxic to animals, and they may be produced by the plant as a chemical defence to discourage grazing by insects and other animals (including goats!). Complex I is a remarkable multiprotein machine that is embedded in the mitochondrial inner membrane and contains over 40 subunits [132], and it perhaps makes a good target for such defensive compounds because of its crucial role in metabolism, and because many hydrophobic molecules might find inhibitory binding sites within such a complex structure. The plant cells that produce these compounds are tolerant to them, because they are usually transported to, and stored in, either the apoplast (the extracellular space containing the cell walls) or the *vacuole*, where they would not come into contact with the mitochondria of the plant cells themselves [133].

Another interesting feature of some of these compounds (e.g. berberine, metformin, phenformin, galegine) is that they are *cations* (i.e. positively charged), which means that they would accumulate to higher concentrations in the cytoplasm compared with the extracellular space, and then in mitochondria compared with the cytoplasm, because of the membrane potentials (positive outside) across the cell membrane and the mitochondrial inner membrane. It has been pointed out that the overall concentrating effect could be as much as 1000-fold, and that if these compounds were to cause a severe inhibition of the respiratory chain, the mitochondrial membrane potential would fall and their accumulation in mitochondria would be reduced, an intriguing self-limiting mechanism [126].

Although the mechanism by which metformin activates AMPK is now clear, the question as to whether AMPK is responsible for the therapeutic benefits of the drug is still being debated. Indeed, AMPK appears not to be responsible for the short-term effects of metformin to reduce liver glucose output [134], which may be due, instead, to the inhibition of one of the key enzymes of *gluconeogenesis*, FBPase, by increases in the AMP : ATP ratio (see §1). However, through its ability to reduce fat storage, AMPK does appear to be responsible for the effects of metformin to reverse liver *insulin resistance*, thus enhancing the ability of insulin to repress liver glucose output in the longer term [135].

As discussed above, Type 2 diabetes is due to a partial failure of insulin not only to repress glucose production by the liver but also to promote glucose uptake by muscle. AMPK activation activates muscle glucose uptake by promoting translocation of the glucose transporter GLUT4 to the cell membrane [92], but metformin does not appear to be taken up by muscle and, therefore, does not significantly activate AMPK in that tissue. Drugs that activate muscle AMPK might, therefore, have benefits over and above those of metformin. As discussed in §4.5, AMPK contains a unique binding site for allosteric activators called the ADaM site. Several pharmaceutical and biotechnology companies have carried out high-throughput screens and identified potent allosteric activators of AMPK that bind this site. Many are selective activators of AMPK complexes containing the *β*1 isoform and these do not activate AMPK in muscle, which primarily expresses the *β*2 isoform [136]. However, two companies have recently reported on the development of ‘pan-β’ activators that activate AMPK complexes containing either *β* isoform, and these have been found to be effective in lowering blood glucose in both rodent and non-human primate models of Type 2 diabetes [136,137]. As yet, these have not been tested in humans, but due to their actions primarily being on the muscle, it would be expected that their effects should be additive with those of metformin.

As discussed in §4.5, AMPK is also activated by binding of salicylate to the ADaM site [48]. Salicylate can be taken orally in the form of acetyl salicylate (aspirin) and salsalate (a dimeric salicylate ester) both of which are rapidly broken down to salicylate when they enter the bloodstream. Interestingly, in human clinical trials to treat obese individuals who were in a pre-diabetic state (i.e. with elevated blood glucose but below the threshold to be classified as diabetic), salsalate was found to be more effective in lowering blood glucose than placebo [138]. Although it was proposed that this effect was due to the inhibition of another signalling pathway, the concentrations of salicylate measured in the bloodstream should have been sufficient to cause activation of AMPK [48], and this may have contributed to the glucose-lowering effect.

The discovery that LKB1 was the principal upstream kinase phosphorylating Thr172 on AMPK [30–32] was exciting, because LKB1 had been previously identified as a *tumour suppressor*, i.e. a protein that restrains cell division and hence cancer [139]. As AMPK activation inhibits cell growth and division (see §6.2 and 6.3), it seems possible that AMPK exerts some of the tumour suppressor effects of LKB1. There is indeed evidence, based on a mouse model of B-cell lymphoma, that AMPK exerts tumour suppressor effects by switching metabolism from the glycolytic mode (typical of many tumour cells) to the more oxidative mode (typical of quiescent cells [140]). However, the role of AMPK in cancer is complex. In proliferative disorders of blood cells, such as leukaemias and lymphomas, it is perhaps less likely that the cells will become starved of nutrients or oxygen, and AMPK may indeed suppress growth of the cancer cells and have anti-cancer effects. However, in solid tumours, where tumours may grow so rapidly that they exceed the capacity of existing blood vessels to supply them with oxygen and nutrients, AMPK may paradoxically aid survival of cells that are under nutrient stress and, as discussed in §4.6, may even improve their blood supply by promoting *angiogenesis*. Consistent with this, genes encoding the *α*1 and *β*2 isoforms of AMPK are frequently amplified together in cancers, especially in adenocarcinoma of the lung (the commonest form of lung cancer), where the frequency may be as high as 10–15% of cases [27,141]. Our current hypothesis is that, by restraining cell growth and division, over-expression of AMPK may limit growth and thus aid survival of the cells until an improved blood supply has been established by angiogenesis. In such cases, inhibitors of AMPK might provide therapeutic benefits by enhancing the effectiveness of existing chemotherapeutic agents.

## 7. Conclusion

AMPK is expressed universally in all eukaryotes, with the notable exception of certain parasites that mainly reproduce inside other eukaryotic cells, which may have been able to dispense with AMPK, because their host cell would provide it. The classical or canonical role of the AMPK system is a sensor of cellular energy status, and we speculate that it may have evolved quite soon after the endosymbiotic acquisition of aerobic bacteria into an archaeal host cell, which many people believe is the critical event that led to the development of the first eukaryote. As in modern day organisms, AMPK promotes mitochondrial biogenesis when it detects that the primary output of the organelle, ATP, is insufficient to meet demand, it is interesting to speculate that AMPK evolved to provide the key interface between the host cell and its newly acquired endosymbiont.

The AMPK heterotrimer is a complex multiprotein machine that converts changes in cellular adenine nucleotides into changes in protein kinase activity. Structural analysis is slowly revealing how AMPK succeeds in the difficult task of detecting small changes in AMP despite the presence of much higher concentrations of ATP.

Genetic evidence from fungi and plants suggests that one of the primary roles of AMPK is in glucose-sensing, and recent evidence suggests that mammalian AMPK can also sense declining glucose supply by a non-canonical mechanism that is independent of changes in AMP and ATP. Glucose-sensing occurs via a complex mechanism involving the Ragulator complex and the *v-ATPase* at the lysosomal surface. Lack of glucose causes a drop in the binding of FBP to the glycolytic enzyme *aldolase*, which is known to bind to the v-ATPase. This triggers the binding of the Axin : LKB1 complex to the v-ATPase and the Ragulator, triggering phosphorylation and activation of AMPK, which may already be partly located at the lysosomal membrane due to N-myristoylation of the *β* subunit. Elucidation of this mechanism has revealed the close links between the regulation of AMPK and mTORC1, another nutrient-sensing pathway that appears to have arisen very early during eukaryotic evolution.

AMPK achieves the difficult task of recognizing and phosphorylating perhaps a few hundred target serine/threonine residues, out of perhaps around a million in a typical cell, by recognizing the pattern of amino acids around the target. In the context of its role as an energy sensor, AMPK acts to restore energy homeostasis by switching on catabolic pathways that generate ATP, while switching off energy-consuming processes such as cell growth and division, and the firing of action potentials in neurons. In the context of its role as a glucose sensor, AMPK switches on catabolic pathways that use alternate carbon sources, such as fatty acids or glutamine. The AMPK system is a prime target for the development of novel drugs aimed at the treatment of metabolic disorders such as obesity and Type 2 diabetes, as well as cancer.

## Supplementary Material

Glossary

## Endnote

1 Biological terms in italics are defined in the glossary that accompanies this article.

## Data accessibility

This article has no additional data.

## Glossary

2R-ohnologues: related genes or proteins within a genome that arose by the two rounds of whole genome duplication thought to have occurred during vertebrate development
action potentials: waves of depolarization (electrical signals) that pass down the long processes emanating from the cell bodies of neurons, triggered by the opening of *voltage-gated Na^+^ channels*
adenylate kinase: enzyme catalysing a reaction that interconverts adenine nucleotides: 2ADP ↔ ATP + AMP
ADP/ATP carriers: proteins that exchange ATP and ADP across biological membranes
agouti-related protein (AGRP): appetite-enhancing (*orexigenic*) neuropeptide produced in *neurons* of the hypothalamus in the brain
aldolase: enzyme involved in *glycolysis* that catalyses the reaction converting fructose-1,6-phosphate to triose phosphates
allosteric activation: activation of an enzyme by a ligand (usually a small molecule) that binds at a site (the allosteric site) distinct from the catalytic site
AMPK: AMP-activated protein kinase, a protein kinase that is activated by AMP and ADP and inhibited by ATP
angiogenesis: the process by which tissues (including malignant tumours) establish a new blood supply
archaea: domain of life distinct from bacteria and eukarya; cells lack internal membrane organelles and usually inhabit extreme environments
autophagosomes: membrane-bound vesicles that deliver cellular constituents to *lysosomes* for recycling
autophagy: process by which cellular constituents are engulfed in *autophagosomes* and delivered to *lysosomes* for recycling
axon: a long process, down which *action potentials* pass, that connects the cell body of a *neuron* to its target cell
bacteria: domain of life distinct from *archaea* and *eukarya*, which lack internal membrane organelles
biguanide: antidiabetic drug containing two guanidine moieties, e.g. *metformin*
C-terminal end: the end of a protein or peptide that contains a carboxyl group not attached to another amino acid (although often blocked by a modifying group)
calmodulin: small Ca^2+^-binding protein that activates many calmodulin-dependent enzymes when Ca^2+^ is bound to it
catalytic site: binding site for substrates on an enzyme, where the reaction catalysed (i.e. speeded up) takes place
conformation: a specific state of folding of a protein
convergent evolution: form of evolutionary change where structures that were originally not related become more similar, due to similar selective pressures
cytoplasm: the major compartment of *eukaryotic* cells, outside of the *nucleus*
domain (of protein): a region of a protein that folds up independently of other regions
endocytosis: process where regions of the cell membrane pinches off to engulf extracellular contents and bring it into the cell
endoplasmic reticulum: network of branching membrane tubules in eukaryotic cells involved in macromolecule biosynthesis, usually close to the nucleus
endosomes: membrane-bound vesicles in eukaryotic cells that deliver their contents either to the lysosome or back to the cell membrane
endosymbiosis: event in which one organism engulfs another, with the two then living together with mutual benefit
endothelial cells: the cells that line blood vessels
eukarya/eukaryotes: domain of life distinct from *archaea* and *bacteria*, which contain internal membrane-bound structures (*organelles*)
excitable cells: cells such as nerve cells that express voltage-gated Na^+^ channels, which can thus carry action potentials (electrical signals) along their external membranes
exocytosis: fusion of internal membrane vesicles with the external cell membrane, thus delivering their contents to the cell exterior
fructose-1,6-bisphosphatase: enzyme involved in *gluconeogenesis* that catalyses the reaction converting FBP to fructose-6-phosphate
G protein/GTP-binding protein: small signalling protein that binds GTP or GDP and can also catalyse the hydrolysis of GTP to GDP
gluconeogenesis: metabolic pathway in which glucose is generated from other precursors, such as lactate and some amino acids
glutaminolysis: metabolic pathway within mitochondria in which glutamine is converted to 2-oxoglutarate, thus entering the TCA cycle
glycolysis: metabolic pathway within the cytoplasm in which glucose is broken down to pyruvate or lactate
golgi apparatus: stacks of membrane vesicle within cells, involved in biosynthesis and secretion
GTPase-activating protein: a protein that promotes GTP hydrolysis by a G protein
guanine-nucleotide exchange factor: a protein that promotes the dissociation of GDP from a G protein and its replacement by GTP
heterotrimer: a stable complex containing three different protein subunits
heterotrophy: lifestyle in which energy is obtained by oxidizing reduced carbon compounds of organic origin, such as glucose
insulin: hormone involved in maintaining blood glucose levels in vertebrates
insulin resistance: disorder in which tissues become resistant to the action of the hormone *insulin*
kinase domain: the region of a protein kinase that carries the catalytic function
lithoautotrophy: lifestyle in which energy is obtained by oxidizing reduced compounds of mineral origin
lysosomes: membrane vesicles within the cytoplasm with acidic interiors, where macromolecules are broken down and their components recycled
mechanistic target-of-rapamycin: protein kinase that is activated by nutrient availability, promoting cell growth
metformin: dimethylbiguanide, a synthetic *biguanide* drug used to treat Type 2 diabetes
mitochondria: membrane-bound organelles in eukaryotic cells that carry out oxidative metabolism, believed to have been derived by endosymbiosis of oxidative bacteria in archaeal host cell
mitochondrial fission: process in which the network of *mitochondria* in cells is fragmented into smaller segments
mitophagy: process by which small segments of *mitochondria* are delivered to *lysosomes* for degradation and recycling of their components
molecular switch: a biological molecule (usually a protein) that can exist in two distinct, inter-convertible structural states
mTORC1: abbreviation for mechanistic target-of-rapamycin complex-1
N-myristoylation: modification of a protein, usually at the N-terminus, with the 14 carbon saturated fatty acid, myristic acid
N-terminal end: the end of a protein or peptide that contains a amine group not attached to another amino acid (although often blocked by a modifying group)
neurons: nerve cells, cells with long processes (dendrites and axons) that are excitable and can, therefore, carry electrical signals
nucleus: the central membrane-bound region of a eukaryotic cell that contains the cellular DNA
organelles: membrane-bound substructures with distinct functions, which occur within *eukaryotic* cells
paralogues: closely related genes or proteins within a single genome, which have most likely arisen by gene duplication
PGC-1*α*: peroxisome proliferator receptor-γ co-activator-1*α*, a transcriptional co-activator involved in promoting the production of *mitochondria* and their components
phenformin: phenethylbiguanide, a synthetic *biguanide* drug formerly used to treat Type 2 diabetes
phosphate ester: a compound formed by chemical combination of a hydroxyl group and phosphoric acid
phosphofructokinase: enzyme involved in *glycolysis* that catalyses the reaction converting fructose-6-phosphate and ATP to FBP
phospholipid bilayer: a biological membrane that contains two layers of phospholipids, with their hydrophobic tails facing each other and their hydrophilic head groups facing outwards
phosphorylase: enzyme involved in *glycogen breakdown* that uses phosphate to release glucose-1-phosphate from the non-reducing ends of glycogen
photoautotrophy: lifestyle in which energy is obtained by absorbing light and using it to reduce CO_2_ to produce organic molecules
portal vein: blood vessel that connects the gastrointestinal tract to the liver
pro-opiomelanocortin (POMC): precursor of appetite-depressing opioid peptides, produced in certain neurons of the hypothalamus in the brain
protein kinase: enzyme catalysing the transfer of phosphate from ATP to hydroxyl group(s) on target protein(s), forming phosphate ester(s)
protein kinase cascade: signalling pathway in which upstream protein kinases phosphorylate and activate downstream protein kinases, thus conveying a signal
protein phosphatase: enzyme catalysing hydrolytic removal of phosphate group(s) on target protein(s), thus reversing protein kinase action
protein phosphorylation: the process in which protein kinases phosphorylate target proteins
prothrombin: soluble precursor of thrombin, a proteinase that triggers blood clotting and can also act as a hormone
quiescent: term that refers to cells that are in a non-dividing, non-growing state
Ragulator: a multiprotein complex involved in the regulation of the mTORC1 pathway
rapamycin: antibiotic that inhibits cell growth by inhibiting the mTORC1 pathway
reactive oxygen species: products of oxygen metabolism that are highly reactive and can react with proteins, lipids and nucleic acids
Rheb: a small G protein that activates mTORC1 when occupied by GTP
sequence motif: a short stretch of amino acids containing a particular pattern of amino acid types
target protein: a protein that can be modified by a modifying enzyme such as a protein kinase
target protein-binding groove: a groove in the surface of a kinase domain, involved in the binding of the target protein
thrombin: a proteinase that triggers blood clotting and can also act as a hormone, the product of prothrombin breakdown
vacuolar ATPase/v-ATPase: a transmembrane multiprotein complex that acidifies the interior of lysosomes and vacuoles by pumping protons into their interior, driven by ATP hydrolysis
vacuole: larger versions of *lysosomes* in fungal and plant cells, often occupying much of the cell volume—in addition to degradative functions of the lysosome, they are used for osmotic control and nutrient storage
vascular endothelial cell growth factor: a protein released by cells that are short of oxygen, which promotes the growth of new blood vessels that would supply oxygen
VEGF: acronym for *vascular endothelial cell growth factor*
vertebrates: animals with backbones, including fish, amphibian, reptiles, birds and mammals
voltage-gated Na^+^ channels: channels activated by membrane depolarization that allow Na^+^ ions to enter into cells, thus permitting the transmission of electrical signals (*action potentials*) along the membrane
Wnt signalling: a signalling pathway involved in embryonic development
