# Supplementary material for: Keeping the home fires burning: AMP-activated protein kinase
Source: J R Soc Interface. 2018 Jan 17;15(138):20170774. doi: 10.1098/rsif.2017.0774 (PMC5805978; doi:10.1098/rsif.2017.0774)
Supplement: Glossary [file rsif20170774supp1.doc]

GLOSSARY

“Keeping the home fires burning- AMP-activated protein kinase” by D. Grahame Hardie

2R-ohnologues related genes or proteins within a genome that arose by the two rounds of whole genome duplication thought to have occurred during vertebrate development

action potentials waves of depolarisation (electrical signals) that pass down the long processes emanating from the cell bodies of neurones, triggered by the opening of *voltage-gated Na^+^ channels*

adenylate kinase enzyme catalysing a reaction that interconverts adenine nucleotides: 2ADP ↔ ATP + AMP

ADP/ATP carriers proteins that exchange ATP and ADP across biological membranes

agouti-related protein (AGRP) appetite-enhancing *(orexigenic)* neuropeptide produced in *neurones* of the hypothalamus in the brain

aldolase enzyme involved in *glycolysis* that catalyses the reaction converting fructose-1,6-phosphate to triose phosphates

allosteric activation activation of an enzyme by a ligand (usually a small molecule) that binds at a site (the allosteric site) distinct from the catalytic site

AMPK AMP-activated protein kinase, a protein kinase that is activated by AMP and ADP and inhibited by ATP

angiogenesis the process by which tissues (including malignant tumours) establish a new blood supply

archaea domain of life distinct from bacteria and eukarya; cells lack internal membrane organelles and usually inhabit extreme environments

autophagosomes membrane-bound vesicles that deliver cellular constituents to *lysosomes* for recycling

autophagy process by which cellular constituents are engulfed in *autophagosomes* and delivered to *lysosomes* for recycling

axon a long process, down which *action potentials* pass, that connects the cell body of a *neurone* to its target cell

bacteria domain of life distinct from *archaea* and *eukarya*, which lack internal membrane organelles

biguanide antidiabetic drug containing two guanidine moieties, e.g. *metformin*

C-terminal end the end of a protein or peptide that contains a carboxyl group not attached to another amino acid (although often blocked by a modifying group)

calmodulin small Ca^2+^-binding protein that activates many calmodulin-dependent enzymes when Ca^2+^ is bound to it

catalytic site binding site for substrates on an enzyme, where the reaction catalysed (i.e. speeded up) takes place

conformation a specific state of folding of a protein

convergent evolution form of evolutionary change where structures that were originally not related become more similar, due to similar selective pressures

cytoplasm the major compartment of *eukaryotic* cells, outside of the *nucleus*

domain (of protein) a region of a protein that folds up independently of other regions

endocytosis process where regions of the cell membrane pinches off to engulf extracellular contents and bring it into the cell

endoplasmic reticulum network of branching membrane tubules in eukaryotic cells involved in macromolecule biosynthesis, usually close to the nucleus

endosomes membrane-bound vesicles in eukaryotic cells that deliver their contents either to the lysosome, or back to the cell membrane

endosymbiosis event in which one organism engulfs another, with the two then living together with mutual benefit

endothelial cells the cells that line blood vessels

eukarya/eukaryotes domain of life distinct from *archaea* and *bacteria*, which contain internal membrane-bound structures *(organelles)*

excitable cells cells such as nerve cells that express voltage-gated Na^+^ channels, which can thus carry action potentials (electrical signals) along their external membranes

exocytosis fusion of internal membrane vesicles with the external cell membrane, thus delivering their contents to the cell exterior

fructose-1,6-bisphosphatase enzyme involved in *gluconeogenesis* that catalyses the reaction converting fructose-1,6-bisphosphate to fructose-6-phosphate

G protein/GTP-binding protein small signalling protein that binds GTP or GDP and can also catalyse the hydrolysis of GTP to GDP

gluconeogenesis metabolic pathway in which glucose is generated from other precursors, such as lactate and some amino acids

glutaminolysis metabolic pathway within mitochondria in which glutamine is converted to 2-oxoglutarate, thus entering the TCA cycle

glycolysis metabolic pathway within the cytoplasm in which glucose is broken down to pyruvate or lactate

Golgi apparatus stacks of membrane vesicle within cells, involved in biosynthesis and secretion

GTPase-activating protein a protein that promotes GTP hydrolysis by a G protein

guanine nucleotide exchange factor a protein that promotes the dissociation of GDP from a G protein and its replacement by GTP

heterotrimer a stable complex containing three different protein subunits

heterotrophy lifestyle in which energy is obtained by oxidizing reduced carbon compounds of organic origin, such as glucose

insulin hormone involved in maintaining blood glucose levels in vertebrates

insulin resistance disorder in which tissues become resistant to the action of the hormone *insulin*

kinase domain the region of a protein kinase that carries the catalytic function

lithoautotrophy lifestyle in which energy is obtained by oxidizing reduced compounds of mineral origin

lysosomes membrane vesicles within the cytoplasm with acidic interiors, where macromolecules are broken down and their components recycled

mechanistic target-of-rapamycin protein kinase that is activated by nutrient availability, promoting cell growth

metformin dimethylbiguanide, a synthetic *biguanide* drug used to treat Type 2 diabetes

mitochondria membrane-bound organelles in eukaryotic cells that carry out oxidative metabolism, believed to have been derived by endosymbiosis of oxidative bacteria in archaeal host cell

mitochondrial fission process in which the network of *mitochondria* in cells is fragmented into smaller segments

mitophagy process by which small segments of *mitochondria* are delivered to *lysosomes* for degradation and recycling of their components

molecular switch a biological molecule (usually a protein) that can exist in two distinct, inter-convertible structural states

mTORC1 abbreviation for mechanistic target-of-rapamycin complex-1

N-myristoylation modification of a protein, usually at the N-terminus, with the 14 carbon saturated fatty acid, myristic acid

N-terminal end the end of a protein or peptide that contains a amine group not attached to another amino acid (although often blocked by a modifying group)

neurones nerve cells, cells with long processes (dendrites and axons) that are excitable and can therefore carry electrical signals

nucleus the central membrane-bound region of a eukaryotic cell that contains the cellular DNA

organelles membrane-bound substructures with distinct functions, which occur within *eukaryotic* cells

paralogues closely related genes or proteins within a single genome, which have most likely arisen by gene duplication

PGC-1α peroxisome proliferator receptor-γ co-activator-1α, a transcriptional co-activator involved in promoting production of *mitochondria* and their components

phenformin phenethylbiguanide, a synthetic *biguanide* drug formerly used to treat Type 2 diabetes

phosphate ester a compound formed by chemical combination of a hydroxyl group and phosphoric acid

phosphofructokinase enzyme involved in *glycolysis* that catalyses the reaction converting fructose-6-phosphate and ATP to fructose-1,6-bisphosphate

phospholipid bilayer a biological membrane that contains two layers of phospholipids, with their hydrophobic tails facing each other and their hydrophilic head groups facing outwards

phosphorylase enzyme involved in *glycogen breakdown* that uses phosphate to release glucose-1-phosphate from the non-reducing ends of glycogen

photoautotrophy lifestyle in which energy is obtained by absorbing light and using it to reduce CO_2_ to produce organic molecules

portal vein blood vessel that connects the gastrointestinal tract to the liver

pro-opiomelanocortin (POMC) precursor of appetite-depressing opioid peptides, produced in certain neurones of the hypothalamus in the brain

protein kinase enzyme catalysing the transfer of phosphate from ATP to hydroxyl group(s) on target protein(s), forming phosphate ester(s)

protein kinase cascade signalling pathway in which upstream protein kinases phosphorylate and activate downstream protein kinases, thus conveying a signal

protein phosphatase enzyme catalysing hydrolytic removal of phosphate group(s) on target protein(s), thus reversing protein kinase action

protein phosphorylation the process in which protein kinases phosphorylate target proteins

prothrombin soluble precursor of thrombin, a proteinase that triggers blood clotting but can also act as a hormone

quiescent term that refers to cells that are in a non-dividing, non-growing state

Ragulator a multiprotein complex involved in regulation of the mTORC1 pathway

rapamycin antibiotic that inhibits cell growth by inhibiting the mTORC1 pathway

reactive oxygen species products of oxygen metabolism that are highly reactive and can react with proteins, lipids and nucleic acids

Rheb a small G protein that activates mTORC1 when occupied by GTP

sequence motif a short stretch of amino acids containing a particular pattern of amino acid types

target protein a protein that can be modified by a modifying enzyme such as a protein kinase

target protein binding groove a groove in the surface of a kinase domain, involved in the binding of the target protein

thrombin a proteinase that triggers blood clotting but can also acs as a hormone, the product of prothrombin breakdown

vacuolar ATPase/v-ATPase a transmembrane multiprotein complex that acidifies the interior of lysosomes and vacuoles by pumping protons into their interior, driven by ATP hydrolysis

vacuole larger versions of *lysosomes* in fungal and plant cells, often occupying much of the cell volume - in addition to degradative functions of the lysosome, they are used for osmotic control and nutrient storage

vascular endothelial cell growth factor a protein released by cells that are short of oxygen, which promotes the growth of new blood vessels that would supply oxygen

VEGF acronym for *vascular endothelial cell growth factor*

vertebrates animals with backbones, including fish, amphibian, reptiles, birds and mammals

voltage-gated Na^+^ channels channels activated by membrane depolarisation that allow Na+ ions to enter into cells, thus permitting the transmission of electrical signals *(action potentials)* along the membrane

Wnt signalling a signalling pathway involved in embryonic development
